# Supplementary material for: The COMMAND trial of cognitive therapy for harmful compliance with command hallucinations (CTCH): a qualitative study of acceptability and tolerability in the UK
Source: BMJ Open. 2018 Jun 15;8(6):e021657. doi: 10.1136/bmjopen-2018-021657 (PMC6009539; doi:10.1136/bmjopen-2018-021657)
Supplement: Author's manuscript [file bmjopen-2018-021657.draft_revisions.pdf]

BMJ Open is committed to open peer review. As part of this commitment we make the peer review history of every article we publish publicly available.

When an article is published we post the peer reviewers' comments and the authors' responses online. We also post the versions of the paper that were used during peer review. These are the versions that the peer review comments apply to.

The versions of the paper that follow are the versions that were submitted during the peer review process. They are not the versions of record or the final published versions. They should not be cited or distributed as the published version of this manuscript.

BMJ Open is an open access journal and the full, final, typeset and author-corrected version of record of the manuscript is available on our site with no access controls, subscription charges or pay-per-view fees (http://bmjopen.bmj.com).

If you have any questions on BMJ Open's open peer review process please email info.bmjopen@bmj.com

BMJ Open

The COMMAND trial of cognitive therapy for harmful compliance with command hallucinations (CTCH): a study of acceptability and tolerability.

Journal:	*BMJ Open*
Manuscript ID	bmjopen-2018-021657
Article Type:	Research
Date Submitted by the Author:	08-Feb-2018

Complete List of Authors:	Birchwood, Max; University of Warwick, Division of mental health & well-being<br>Mohan, Laura; University of Birmingham, College of Medicine<br>Meaden, Alan; Birmingham and Solihull Mental Health NHS Foundation Trust<br>Tarrier, Nick; University of Manchester<br>Lewis, Shon; University of Manchester<br>Wykes, Til; King's College London (Institute of Psychiatry),<br>Davies, Linda; University of Manchester, Institute of Population Health<br>Peters, Emmanuelle; Kings College, London, IPPN<br>Michail, Maria; University of Birmingham, School of Psychology

Keywords:	Command hallucinations, Psychosis, CBT, Harm

SCHOLARONE™
Manuscripts

The COMMAND trial of cognitive therapy for harmful compliance with command hallucinations (CTCH): a study of acceptability and tolerability.

Max Birchwood*, Laura Mohan, Alan Meaden, Nicholas Tarrier, Shon Lewis, Til Wykes, Linda Davies, Graham Dunn, Emmanuelle Peters and Maria Michail

Warwick Medical School, Mental Health and Wellbeing, University of Warwick, UK (Prof M Birchwood DSc)

College of Medicine and Dentistry, University of Birmingham (L Mohan),

Faculty of Health Science, Birmingham City University, Birmingham, UK (Professor A Meaden, D Clin Psy);

Division of Psychology & Mental Health (Prof Lewis, PhD and Prof Tarrier, PhD.)

School of Psychology, University of Birmingham (M Michail, PhD)

Centre for Biostatistics, Institute of Population Health, University of Manchester, Manchester, UK (Prof G Dunn PhD)

Kings College, Institute of Psychiatry University of London (E Peters PhD, Prof T Wykes, PhD)

* Corresponding author: Professor Birchwood, m.j.birchwood@warwick.ac.uk

For peer review only

Abstract

Objectives: To explore service user experiences of a 9-month cognitive behavioural therapy for command hallucinations in the context of a randomized controlled trial including their views on acceptability and tolerability of the intervention.

Design: Qualitative study using semi-structured interviews.

Setting: The study took place across three sites: Birmingham, Manchester and London. Interviews were carried out at the sites where therapy took place, which included service bases and participants' homes.

Participants: Of 197 patients who consented to the trial, 98 received the CTCH intervention; 25 (15 males) of whom were randomly selected and consented to the qualitative study. The mean age of the sample was 42 years, and 68% were White British.

Results: Two superordinate themes were identified: participants' views about the aspects of CTCH they found most helpful; and, participants' concerns with therapy. Helpful aspects of the therapy included: gaining control over the voices; challenging the power and omniscience of the voices; following a structured approach; normalisation and mainstreaming of the experience of voices; and having peer support alongside the therapy. Concerns with the therapy included anxiety about completing CTCH tasks, fear of talking back to voices, the need for follow up and ongoing support and concerns with adaptability of the therapy.

Conclusions

Interpretation: CTCH was generally well received and the narratives validated the overall approach. Participants did not find it an easy therapy to undertake as they were challenging a persecutor they believed had great power to harm; many were concerned, anxious and occasionally disappointed that the voices did not disappear altogether. The trusting relationship with the therapist was crucial. The need for continued support was expressed.

Strengths and limitations of this study

- The study was presented in line with the Consolidated Criteria for Reporting Qualitative Research (COREQ) ensuring the explicit and comprehensive reporting of all study elements.
- Interviews and analyses were conducted by team members not involved in the delivery of the intervention or data collection for the larger RCT reducing potential bias.
- The absence of participants who did not engage in therapy at all or dropped out as their views would have been valuable in highlighting difficulties with engagement and reasons for withdrawing from CTCH.

For peer review only

Background

Acting on command hallucinations in psychosis can have serious consequences for the individual and for other people and is a major cause of clinical and public concern. Many individuals who experience auditory hallucinations include commands that instruct them to do harm to self, others or objects [(1) (2)]. 53% of a sample of adult psychiatric patients continue to hear voices; of these, 48% stipulated harmful or dangerous actions rising to 69% among patients in medium secure units [(3)] as reported in Shawyer et al. [(1)] While it is difficult to predict individual acts of compliance, those who have complied with voices with serious harm to themselves or others within the previous 9 months are at high risk of repeating this in the following 18 months. [(4)]

Based on our cognitive model of voices, we have shown that it is not only the level of activity of voices, or indeed their content that drives affect and behaviour, but the nature of the relationship with the personified voice. [(6)(7)(8)] We showed that where the voice hearer believes the voice to have malevolent intent, and crucially to have the power to deliver the threat, this can motivate compliance or appeasement behavior. [(9)] These findings have been independently replicated in a forensic population. [(10)] This theoretical framework informed the development of our Cognitive Behaviour Therapy for Command Hallucinations (CTCH) designed to weaken and challenge beliefs about voices' power, thus enabling the individual to break free of the need to comply or appease and thereby reduce harmful compliance behavior and distress. [(10)(11)]

We completed the first major trial of CTCH focusing on this dimension of the voice experience, the perceived power of the voice, to reduce the motivation to comply. [(12)] The trial demonstrated that the risk of further serious compliance was high in the treatment as usual group (46%), reducing to 28% in those receiving the CTCH. The rate of consent to the trial and adherence to the CTCH were in excess of 80%, providing indirect evidence that the therapy was needed and was acceptable.

The aim of this study was to explore service user experiences of a 9-month CTCH in the context of a randomized controlled trial (SRCTN62304114) including their views on acceptability and tolerability of the intervention.

Methods

Study design and settings

This was a qualitative study embedded within a larger RCT comparing CTCH and treatment as usual with treatment as usual alone. The trial involved 197 eligible participants from 3 UK centres. Participants were assigned to the intervention or control arm of the trial in a 1:1 ratio. The treatment period was a maximum of 9 months and follow up occurred at 9 and 18 months after randomization. Participants were eligible if they met all of the following criteria: had International Statistical Classification of Diseases and Related Health Problems, 10th Revision (ICD-10), schizophrenia, schizoaffective (F20, 22, 23, 25, 28, 29), or mood disorders (F32),19 and were under the care of a clinical team; were aged 16 years and older; had a history of harmful command hallucinations for at least 6 months with recent (< 9 months) history of harm to self or others, or major social transgressions as a result of the commands (full or incomplete compliance); or had harmful command hallucinations whereby the individual was distressed and appeasing the powerful voice. Individuals with a primary diagnosis of addictive disorder, an organic impairment or

For peer review only - http://bmjopen.bmj.com/site/about/guidelines.xhtml

insufficient command of English were excluded from the study. The protocol for the cognitive therapy, described in Meaden et al [11] allows the treatment to be tailored to the individual. The intervention was described in the main trial publication [12], with illustrative cases written up in our manual [13][14]Ethical approval for the study was provided by The West Midlands Research Ethics Committee (number 06/MRE07/71).

Research team and reflexivity

The interviews were conducted by one of the co-applicants (HL) and two research assistants (KP and LB), all unknown to participants, and examined the thoughts, concerns and experiences of the CTCH in an open manner. Therapists did not conduct the interviews nor were they present for them. The interviews continued until the participant felt they had nothing more to say in response to the framework questions proposed to them (probe questions). The interviews each lasted for approximately half an hour. Interviews were coded by two raters not involved in the trial. We believe therefore that the data collected here were representative of those receiving therapy and interpreted without bias.

Sample

A sample of 25 participants was drawn, who were selected randomly by number generator, from the total of 98 receiving the therapy in the trial (25.5%). These included 8 from the Birmingham site, 10 from London and 7 from Manchester, with a range of ages and genders included. The sample included participants who had completed all of the sessions as part of the CTCH program, and who consented to be interviewed and recorded for the purposes of this study. Interviews were carried out until theoretical saturation was achieved with no new themes emerging; this was achieved at 25 interviews. Participants were assured that the interviews would be anonymous and individual views of therapists would not be communicated to the therapists.

Data collection and analysis

Face-to-face semi-structured qualitative interviews were carried out at the sites where therapy took place, which included service bases and participants' homes. Each interview was audio recorded, transcribed verbatim (by PRSL) and checked for accuracy. Personal details were removed to preserve participant anonymity. Field notes were written in the form of memos. The study is presented in line with the Consolidated Criteria for Reporting Qualitative Research (COREQ).

The interviews were structured but flexible in allowing follow up to participant supplied information. They focused on a brief history of the patients' difficulties and symptoms, followed by discussion of the therapy they had received, and the positive and negative aspects of receiving the therapy as felt by the participants. The interview guide included the following probe questions:

Do you feel that the therapy helped you? Why do you feel that that session (which participant mentioned) was (un)helpful for you?

Was the therapy acceptable for you?

How was your relationship with [the therapist]?

Did you feel the therapist understood what it was like to hear voices?

For peer review only - http://bmjopen.bmj.com/site/about/guidelines.xhtml

How was the length of the sessions and the program as a whole?

What was the most helpful session? What was the least helpful session?

Do you feel the therapy made a difference at all?

The interviews were analysed using adapted grounded theory [15]. Theories were generated from the data. Evidence of dissonance was sought throughout and theories were altered to accommodate this. Quotations have been chosen to be representative of the data collected. Each transcript was read and reread by LM and EE, until saturation of data was reached, and no new themes emerged.

There was line by line thematic analysis and discussion on any discourse present in the analysis of the themes. Words, ideas and reported experiences in the participants' texts was completed with an inductive approach and no pre-existing framework for the ideas. Agreement between LM and EB was high when it was discussed at regular meetings. Thematic coding was grouped into key emergent themes, which we present as findings in this paper.

Findings

The sample consisted of 25 participants, 15 of whom were males. The mean age of the sample was 42 years, and 68% were White British. Thirty-six per cent (36%) of the sample was diagnosed as 'unspecified psychosis', 56% as 'Schizophrenia' and 8% as Schizo-affective disorder. The demographic characteristics of the sample are presented in Table 1.

Table 1 about here.

Two superordinate themes were identified: participants' views about the aspects of CTCH they found most helpful and participants' concerns with therapy. The themes (and sub-themes) are presented below and supported by illustrative quotations from the transcripts.

Helpful aspects of CTCH

The components of CTCH participants reported finding most helpful in tackling their mental health problems were related to:

(i) Feeling more in control

Several individuals discussed one of the outcomes of the therapy as gaining more control over the voices and being able to cope better with their mental health problems and the impact these had on their lives. Gaining control over the voices was one of the main components and aims of the intervention and in our previous work we were able to show that participants who received the CTCH intervention felt more powerful, than those in the control group, in their ability to withstand and mitigate the threat received by their voices.

'I'm able to control them [the voices] a bit more.' (F52BIR104)

"I cope better now because I listen to what [the therapist] said." (F67LON013)

For peer review only - http://bmjopen.bmj.com/site/about/guidelines.xhtml

"I feel like I've got control of them [the voices]." (M30BIR125)

(ii) Challenging the power and omniscience of the voices

Participants spoke about how being encouraged to reflect on evidence for voices' power and omniscience particularly helped them during the therapy. Using examples from everyday life, therapists and participants worked collaboratively to show that voices made empty threats which subsequently led participants to realise that "*the problems are all inside me. They are not out there*", as one participant noted. The therapy aimed to build on participants' own strengths to face up to the voices, and so make the voices less fearful. As indicated below, this technique was reported as particularly useful by the participants.

'She said, if we put the papers down and let's see if they come in and take them, and I said they didn't. [In response to patients belief that the voices would take her therapy notes from the therapist to find out what she had told others of the voices] I think that helped, you know. It just gives me a bit of support that they're lying. They are just talking.' (F67LON013)

'She [the therapist] was pregnant and [the voice] says that something will happen to her baby. And she had the baby and nothing happened, and that was really a good reassurance that [the voice] is just in my mind and not real. [The therapist] says he [the voice] could bring it on, that it won't hurt her and she was sure the baby would be alright. I know he can't do things physically, just mentally.' (F57BIR094)

'[The voice] is just in my mind and not real.' (F57BIR094)

(iii) Following a structured approach

Participants described how taking a structured approach to the therapy sessions helped them get an overview of the therapy in the context of a complex and confusing experience. Some participants specifically commented that understanding the triggers for their voices helped to gain mastery, as illustrated by the quotes below.

'It was very structured; all the time we had an agenda which was good' (F42LON077)

'She gave me some information afterwards…I could remember the session where she was helping me through this.' (F48LON085)

"Connecting things together…change your pattern and change your ways.' (F56LON009)

'She drew me a diagram between the bad thoughts and the good thoughts, and how the brain works and that diagram was helpful.' (F48LON085)

(iv) Normalisation and mainstreaming of the experience of voices

For peer review only - http://bmjopen.bmj.com/site/about/guidelines.xhtml

Many participants described how the acceptance of voice hearing reduced feelings of isolation and demonstrated through the experience of others that control was possible. Normalisation of the voice appeared to reduce the ‘specialness’ of the voice, as well as offering support to the voice hearer. Participants commented how finding out that hearing voices is common and “*can happen to most people*” (M25MAN062) was empowering and helped them feel better about themselves.

‘[The therapist] was like ‘oh it’s common and can happen to most people.’ I wasn’t aware of that… She was telling me how the voices can be controlling and stuff.’ (M25MAN062)

‘[watched a DVD called ‘am I normal’ by the BBC, suggested by therapist] people that either hear voices or have heard voices and they’re back in work, or they’re living some kind of normal life or they’re in respected positions, but they still hear voices. That was helpful; let me feel that I wasn’t alone. I felt a little bit better in myself.’ (M53LON001)

(v) The value of peer support

Participants highlighted that provision of peer support alongside the therapy was beneficial in normalizing their experiences, supporting them with challenging or high risk situations such as going on assignments outside, and encouraging them in engaging with difficult aspects of the therapy such as challenging the power of voices. In particular, participants felt that peer support helped them feel included and listened to; empowered them to help others, or as one participant said “give something back”; and also benefited their social life.

‘The family and people where I’m living now in support housing [listen to me]. We talk about the voices. They listen to me and talk to me like, saying things aren’t going to happen while you’re living here.’ (M30BIR126)

‘I’m going to a place on Tuesday, and you talk about the issues you wanna talk about. They’re there to listen.’ (M41LON101)

‘She [the therapist] put me in touch with the healing arts team, and another place called [an arts based support group for people with mental health issues] …a couple of times we went outside.’ (M53LON072)

[Patient in conversation with another patient with psychosis] ‘I just told her it gets better and he won’t win. Even though you won’t feel like it now, he won’t win. [Interviewer asks if she recommended speaking back to the voices, patient responds] ‘yeah tell them to get lost.’ (F25MAN067)

‘I really wanna do something, like give back what they give you. Take it and help other people.’ (F40MAN079)

‘I [the participant] gave her [the therapist] an example on how to cope with voices.’(M37BIR013)

Concerns with therapy

For peer review only - http://bmjopen.bmj.com/site/about/guidelines.xhtml

Participants also expressed several concerns with the therapy. These were mainly associated with the experience of persecutory feelings interfering with the therapy process, for example concerns about the therapist and their intentions; but also receptiveness to accepting psychological therapy. Adapting the therapy to the individual and reassurance regarding the lack of real world power of the voices were reported as helping with the tolerability of the therapy.

(i) Fear of therapy

Many participants described fear of the therapy arising from a perceived lack of ability to perform the tasks requested as part of the therapy, such as talking back to the voices. This could be related to lack of self–confidence which seemed to be more an issue at the early stages of the therapy yet caused significant distress to participants. A commonly reported concern was talking about the voices to the therapist due to fear about what the voices might do or say. For some individuals, the voices spoke directly about the therapist and instructed the individual not to reveal their conversations.

'I was frightened about starting the therapy, because I didn't know quite what to expect…. [During the therapy] I felt unsure whether I was answering them [the questions] correctly.' (F48LON085)

'They tell you not to talk about them…so you're frightened to talk to someone about it.' (F48LON100)

'It's quite frightening to try and explain these voices.' (F67LON013)

(ii) Challenges to established patterns of thinking and behaviour

Some participants described feeling upset at times when asked to think through and challenge long-established beliefs about their voices and their behaviour in response to the voices. For example, one participant described finding it very difficult to talk back to the voices following the therapist's suggestion. Trying out new ways of coping, for example testing the voice as a way of challenging participants' entrenched beliefs about the voices and their associated power was also something participants found challenging. In some cases there was resistance to some of the new suggestions and ways of coping. One participant said:

"'I got my own way of coping with things and she was trying to introduce new ways and I found it hard to try to practice them coz I was used to thinking a certain way.' (M50MAN081)"

This resistance could be related to perceived lack of self-confidence and the fear of therapy discussed previously but also highlights other concerns such as participants' fear at changing the strategies they already have for dealing with the voices. Personalising aspects of the therapy to address such concerns would be necessary, as therapy needs to take into account an individual's beliefs, fears and coping methods to plan a way forward.

'It was upsetting at times, I would leave here in floods of tears at times, but I carried on.'(F48LON085)

'[the therapist suggested the patient] should talk back to them, try and reason with them, but I didn't think that was a very good idea because then I'm being pulled into their games.' (F27LON105).

For peer review only - http://bmjopen.bmj.com/site/about/guidelines.xhtml

(iii) Building trust with the therapist

Many participants described how building a trusting relationship with the therapist was difficult as new and sometimes challenging ideas were discussed during therapy which some individuals felt placed them at risk from the voices. Building trust with the therapist was perceived by participants to be important in allowing them to feel confident to change established patterns of thinking and behavior. Participants explained how this did not happen overnight and a collaborative effort from the therapist and individual was necessary to achieve that. Some described how their therapist worked practically alongside them in a facilitating role, while others discussed having the therapy in an environment where they felt safe.

'It took me a little while to build up the trust [with the therapist].' (F48LON100).

'I was very frightened about leaving the hospital and on just one occasion she came and took the bus with me part way. She walked me to the bus and got on the bus with me, and I found that very supportive, and it gave me the encouragement to be able to leave the session and do it on my own. ' (F42LON077).

'She [the therapist] helped me start going places where there's other people.' (F25MAN067)

'And then we started going [to have the therapy at] my Grans and it was alright…. Because I feel safe at my Grans.' (F25MAN067)

(iv) Need for better personalisation of therapy

While some participants were pleased that the therapy and the therapists were sensitive to the individual and their circumstances, including any fears and concerns, others voiced concerns that the therapy was not adaptable enough and provided some examples of how the content, duration and tasks of the therapy could be tailored to the individual's needs and preferences.

'It [the therapy] should have gone to a practical therapy. Actually confront the fears together and she could go through what I was thinking at certain points. Actually go to the public places.' (M50MAN081).

'[in response to is there anything you would change] The sessions should last longer sometimes. Or shorter sometimes. [So be more flexible?] Yeah. It was how much it applied to you.' (M46MAN076)

'She gave me things to do, like do you have any hobbies or something to take your mind off…. I was looking at a book I just couldn't read…. She said to make some noise like 'mmmmh' to like, to stop it……I felt silly doing that in the living room and my parents were [enquiring why the patient was making the noise]'(M25MAN062)

(v) Expectations of therapy: control vs cure

Participants' expectations of therapy i.e. their beliefs about the consequences of receiving treatment, such as that the treatment will lead to improvement, was a recurrent issue. Some participants reported being disappointed that the therapy did not have the impact they had

For peer review only - http://bmjopen.bmj.com/site/about/guidelines.xhtml

expected, for example, it did not make the voices go away or as one participant said '*the magic never seemed to start*'.

'I just kept thinking, when's the magic going to start.' (M50MAN081)

'They've [the voices have] not gone away completely. You know unfortunately I thought they would but they haven't.' (F42LON077)

Discussion

In this study, we explored service user experiences of a 9-month cognitive behavioural therapy for command hallucinations in the context of a randomized controlled trial including their views on acceptability and tolerability of the intervention.

The study was presented in line with COREQ ensuring the comprehensive reporting of all study elements. Interviews and analyses were conducted by team members not involved in the delivery of the intervention or data collection for the larger RCT. Indeed, therapists were not involved in the qualitative study nor were present during the interviews. A limitation of this study was the absence of participants who did not engage in therapy at all or dropped out as their views would be valuable in highlighting difficulties with engagement and reasons for withdrawing from CTCH.

Few CBT studies in psychosis have sought systematically to document participants' experience of therapy [5] [16]. A recent synthesis of existing qualitative studies concluded that an alliance between service user and therapist, clear steps in facilitating change, and the challenges of applying CBTp (cognitive behavioral therapy for psychosis) are the main reported experiences of users [5]. In this study too, participants highlighted the importance of the therapist-service user relationship in distancing from voices' power, the value of structure in the therapy, and how the CTCH could be difficult and distressing for participants. In addition, the present study also documented how normalising the voice experience served to gain intellectual mastery of it (i.e. voices were no longer 'special' or 'mysterious') and many highlighted the need for longer-term, preferably and peer support.

The components of the CTCH service users found most useful was gaining more control over the voices and challenging the power of voices to act on their supposed malevolent intent. The words 'control' and 'cope' cropped up frequently in the narratives of participants. Many valued the structured approach to examining long-held beliefs and practices; for example clarity about the triggers for voices helped to achieve intellectual mastery of the experience and as a basis to challenge the power of the persecutor behind the voice. Others described the value in normalising the experience; this characterisation as an everyday event served as an antidote to the 'special' place of the voice and indeed of the voice hearer within it. Some argued that this could have gone further with improved peer support and validation. Trusting the therapist was brought up in many of the narratives. This is understandable from the client's point of view as from their perspective, the voice is embodied with major threats, in some cases life-threatening, and hence trusting the therapist's motives and ability was seen as crucial. Participants recounted many examples of the

For peer review only - http://bmjopen.bmj.com/site/about/guidelines.xhtml

lengths therapists went to achieve this; accompanying them on assignments in supposed high risk situations was often described and valued by the participants.

Some concerns with the therapy, albeit minor, were also expressed and these centered on the experience of persecutory feelings interfering with the therapy process, for example concerns about the therapist and their intentions; but also receptiveness to accepting psychological therapy. Many participants reported feeling fearful of the therapy and found the process of facing up to the power of voices at times distressing. Simply articulating these complex experiences was sometimes profoundly difficult for individuals. In some cases, the voices attempted to exert counter-control by questioning the therapist and commanding the individual to disengage. This is perhaps where the suggestion for more peer support from participants would have been beneficial. It was also noted to be beneficial for the therapist to provide reassurance in the form of reiterating that the voices cannot cause affect in the real world. Nonetheless, none of the individuals we spoke to expressed strong adverse experiences of the therapy and as we highlight, few dropped out.

Implications for practice

The views of participants captured in this study combined with the high level of completion of the therapy and retention in the trial, indicates that the CTCH is an acceptable intervention and generally well tolerated. Clinicians working with individuals experiencing harmful voices should be mindful of asking, as part of their assessment, about the perceived power of the voice relative to the individual. This is important as our previous work [17] has shown that the perceived power of voices to threaten the individual, was the best predictor of harmful compliance to voices.

Nonetheless, clinicians should also be mindful of challenges reported by individuals in relation to the CTCH e.g. not being an easy therapy to undertake; fear that the approach could increase the perceived threat from voices, and maximize efforts to build alliance. We recommend peer-support as a useful addition to this therapy, particularly in providing support following its conclusion but also in providing help during difficult periods when the voice's power is confronted and to further normalize the experience (an approach recently highlighted as a *'promising practice'* in the 2014 NICE guidance for Schizophrenia). [18] We also recommend to therapists implementing this approach to make it clear that the intervention is not designed to eliminate voices rather build on individuals' own strengths to face up to the power of the voice, to make them less fearful and place them in the 'driving seat'.

For peer review only - http://bmjopen.bmj.com/site/about/guidelines.xhtml

Funding: This work was supported by the UK Medical Research Council (G0500965) and the National Institute for Health Research.

Competing interests: None declared.

Patient consent: Obtained.

Contributors: MB, EP, SL, TW, NT, LD, and GD designed the randomized controlled trial. MM was the trial manager. MB developed the cognitive therapy for command hallucinations. MB and AM designed and led the training for cognitive therapy for command hallucinations, and AM and EP led the supervision process and the rating of therapy tapes. HL conducted all qualitative interviews. LM and EG analysed the data and wrote the first draft of the manuscript. LM and MM redrafted the manuscript. All authors read and approved the final manuscript. MB had full access to all the data in the study and had final responsibility for the decision to submit for publication.

Data sharing statement: Additional data such as anonymised transcripts are available by emailing MB on m.j.birchwood@warwick.ac.uk

For peer review only

For peer review only - http://bmjopen.bmj.com/site/about/guidelines.xhtml

References

1. Shawyer F, Mackinnon A, Farhall J, Trauer T, Copolov D. (2003). Command hallucinations and violence: implications for detention and treatment. *Psychiatry, Psychology and Law*, 10:97-107.
2. Appelbaum PS, Robbins P., Monahan J. (2000). Violence and delusions: data from the MacArthur Violence Risk Assesment. *American Journal of Psychiatry,* 157, 566-572
3. Chadwick, P., Birchwood, M. (1994). The omnipotence of voices: a cognitive approach to auditory hallucinations. *British Journal of Psychiatry*, 164*,* 190-201.
4. Trower P, Birchwood M, Meaden A, Byrne S, Nelson A, Ross K. (2004). Cognitive therapy for command hallucinations: randomised controlled trial. *British Journal of Psychiatry,* 184 (4) 312-320;
5. Wood L., Burke E., Morrison A, (2015). Individual Cognitive Behavioural Therapy for Psychosis (CBTp): A Systematic Review of Qualitative Literature *Behavioural and Cognitive Psychotherapy*, 43, 285–297
6. Birchwood, M., Gilbert, P., Gilbert, J., Trower, P., Meaden, A., Hay, J., Murray, E. Miles, J.N.V. (2004). Do interpersonal schema influence the relationship with the dominant 'voice' in schizophrenia? A comparison of three models. *Psychological Medicine,* 34, 1571-1580.
7. Connor, C., Birchwood, M. (2013). Power and perceived expressed emotion of voices: their impact on depression and suicidal thinking in those that hear voices. *Clinical Psychology and Psychotherapy, 20(3):199-205.*
8. Byrne S, Trower P, Birchwood M. (2003). Command Hallucinations: cognitive theory, cognitive therapy and research. *Journal of Cognitive Psychotherapy*, 67-84.
9. National Institute for Clinical Excellence. *Schizophrenia*: *Core interventions in the treatment and management of schizophrenia in adults in primary and secondary care*. 1.3.4 Psychological and psychosocial intervention, NICE clinical guideline 82, Level 1A, City Tower, Piccadilly Plaza, Manchester M1 4BT, Issued: March 2009.
10. Fox J, Gray N, Lewis N. (2004). Factors determining compliance with violent content: the role of social rank, perceived power of the voice and voice malevolence. *Journal of Forensic Psychiatry and Psychology,* 15:511-31.
11. Meaden A, Keen N, Aston R, Barton K, Bucci S. *Cognitive Therapy for Command Hallucinations: An advanced practical companion*. Routledge Taylor and Francis Group, London and New York, 2013,
12. Birchwood M, Michail M, Meaden A, Tarrier N, Lewis S, Wykes T et al (2014). Cognitive behavioural therapy to prevent harmful compliance with command hallucinations (COMMAND): a randomised controlled trial. *Lancet Psychiatry*, 1: 23-33.
13. Byrne S, Birchwood M, Trower P, Meaden A. *A Casebook of cognitive behaviour therapy for command hallucinations. A social rank theory approach*. London & New York: Routledge; 2006.

For peer review only - http://bmjopen.bmj.com/site/about/guidelines.xhtml

14. Meaden, A., Keen, N., Aston, R., Barton, K., Bucci, S. *Cognitive therapy for command hallucinations: An advanced practical companion.* 2013 Routledge, Hove.
15. Birks M, Mills J. *Grounded Theory: A Practical Guide*, Sage 2011, London.
16. Kilbride M., Byrne R., Price J., Wood L., Barratt S., Welford M., Morrison A.P. (2012). Exploring service users' perceptions of cognitive behavioural therapy for psychosis: a user led study. *Behavioural and Cognitive Psychotherapy*, 41 (1), 89-102
17. Birchwood M., Dunn G., Meaden A., Tarier N., Lewis S., Wykes T., Davies L., Michail M., Peters E. (2017). The COMMAND trial of cognitive therapy to prevent harmful compliance with command hallucinations: predictors of outcome and mediators of change. *Psychological Medicine*. https://doi.org/10.1017/S0033291717003488
18. National Institute for Clinical Excellence. *Psychosis and schizophrenia in adults: prevention and management,* NICE guidelines [CG178], February 2014

For peer review only - http://bmjopen.bmj.com/site/about/guidelines.xhtml

Table 1. Participant characteristics.

Identifier	Gender	Age	Ethnicity	Diagnosis
F56LON009	Female	56	British	Unspecified psychosis
F67LON013	Female	67	British	Unspecified psychosis
M53LON072	Male	53	Other-white background	Schizophrenia
F42LON077	Female	42	Caribbean	Schizo-affective disorder
F48LON085	Female	48	British	Unspecified psychosis
M18LON099	Male	18	British	Unspecified psychosis
F48LON100	Female	48	British	Unspecified psychosis
M41LON101	Male	41	British	Schizophrenia
F27LON105	Female	27	British	Schizophrenia
M53LON001	Male	53	Otherblack background	Schizophrenia
M39BIR093	Male	39	British	Schizophrenia
F57BIR094	Female	57	British	Schizophrenia
M55BIR016	Male	55	Irish	Schizophrenia
M30BIR125	Male	30	Caribbean	Unspecified psychosis
M35BIR126	Male	35	British	Unspecified psychosis
M37BIR013	Male	37	Caribbean	Schizophrenia
F52BIR104	Female	52	British	Schizo-affective disorder
M34BIR045	Male	34	British	Schizophrenia
F25MAN067	Female	25	British	Unspecified psychosis
M25MAN062	Male	25	British	Schizophrenia

For peer review only - http://bmjopen.bmj.com/site/about/guidelines.xhtml

M31MAN066	Male	31	British	Unspecified psychosis
M46MAN076	Male	46	British	Schizophrenia
M47MAN073	Male	47	Other-black background	Schizophrenia
F40MAN079	Female	40	African	Schizophrenia
M50MAN081	Male	50	British	Schizophrenia

For peer review only

For peer review only - http://bmjopen.bmj.com/site/about/guidelines.xhtml

Articles

Cognitive behaviour therapy to prevent harmful compliance with command hallucinations (COMMAND): a randomised controlled trial

Max Birchwood, Maria Michail, Alan Meaden, Nicholas Tarrier, Shon Lewis, Til Wykes, Linda Davies, Graham Dunn, Emmanuelle Peters

Summary

Background Acting on command hallucinations in psychosis can have serious consequences for the individual and for other people and is a major cause of clinical and public concern. No evidence-based treatments are available to reduce this risk behaviour. We therefore tested our new cognitive therapy to challenge the perceived power of voices to inflict harm on the voice hearer if commands are not followed, thereby reducing the hearer's motivation to comply.

Methods In COMMAND, a single-blind, randomised controlled trial, eligible participants from three centres in the UK who had command hallucinations for at least 6 months leading to major episodes of harm to themselves or other people were assigned in a 1: 1 ratio to cognitive therapy for command hallucinations + treatment as usual versus just treatment as usual for 9 months. Only the raters were masked to treatment assignment. The primary outcome was harmful compliance. Analysis was by intention to treat. The trial is registered, number ISRCTN62304114.

Findings 98 (50%) of 197 participants were assigned to cognitive therapy for command hallucinations + treatment as usual and 99 (50%) to treatment as usual. At 18 months, 39 (46%) of 85 participants in the treatment as usual group fully complied with the voices compared with 22 (28%) of 79 in the cognitive therapy for command hallucinations + treatment as usual group (odds ratio 0·45, 95% CI 0·23–0·88, p=0·021). At 9 months the treatment effect was not significant (0·74, 0·40–1·39, p=0·353). However, the treatment by follow-up interaction was not significant and the treatment effect common to both follow-up points was 0·57 (0·33–0·98, p=0·042).

Interpretation This is the first trial to show a clinically meaningful reduction in risk behaviour associated with commanding voices. We will next determine if change in power was the mediator of change. Further more complex trials are needed to identify the most influential components of the treatment in reducing power and compliance.

Funding UK Medical Research Council and the National Institute for Health Research.

Lancet Psychiatry 2014; 1: 23–33

See Comment page 3

Division of Health and Wellbeing, Warwick Medical School, University of Warwick, Coventry, UK (Prof M Birchwood DSc); Institute of Mental Health, School of Nursing, Midwifery and Physiotherapy, Faculty of Medicine and Health Sciences, University of Nottingham, Nottingham, UK (M Michail PhD); School of Psychology, University of Birmingham, Birmingham, UK (A Meaden DClinPsy); Institute of Brain Behaviour and Mental Health, School of Medicine, University of Manchester, Manchester, UK (Prof S Lewis PhD); Institute of Population Health, University of Manchester, Manchester, UK (Prof G Dunn PhD, Prof L Davies PhD); and King's College London, Institute of Psychiatry, Department of Psychology, London, UK (Prof N Tarrier PhD, Prof T Wykes PhD, E Peters PhD)

Correspondence to: Prof Max Birchwood, Division of Health and Wellbeing, Warwick Medical School, University of Warwick, Coventry CV4 7AL, UK m.j.birchwood@warwick.ac.uk

Introduction

Schizophrenia affects 0·8% of the UK population, usually starts in young adults, and, in some cases, leads to persistent disability.[1] Individuals with this illness have a high risk of suicide (8%) and deliberate self-harm and, although the risk is small, they are more likely to perpetrate aggression than are those without schizophrenia.[2] Individuals acting on delusions, including commanding voices, are a cause for concern at societal and political levels because members of the public are at risk of apparently random acts of violence, even when the perpetrators are well supported by services. These concerns are shown in national policy documents—eg, the UK national mental health strategy aims to reduce avoidable harm to self or others.[3]

Although drug and other treatments have improved, nearly 50% of individuals will have treatment-resistant symptoms or symptoms arising from non-adherence to drug regimens.[4,5]

Auditory hallucinations are some of the most prominent and distressing of the treatment-resistant symptoms, and command hallucinations are the most high risk of these.[6] Shawyer and colleagues[6] reported a median 53% prevalence of command hallucinations in adult participants with psychiatric disorders; 48% of these participants said the commands stipulated harmful or dangerous actions, rising to 69% for participants in medium secure units.[7]

However, the link between the presence of command hallucinations and harm to self or others is not straightforward. In the MaCarthur study,[8] no association was reported between the presence of delusions or command hallucinations and violence. Thoughts about violence, however, were a strong predictor of violence 6 months later.

Our cognitive model of voices has clarified that it is not only the level of activity of voices, or indeed their content, that drives affect and behaviour, but also the nature of the relationship with the personified voice.[9–11] We showed that compliance or appeasement behaviour can occur when the hearer believes the voice to have malevolent intent, and crucially to have the power to deliver the threat.[9] These findings have been independently replicated in a forensic population.[12]

This theoretical framework informed the development of a cognitive behaviour therapy: cognitive therapy for

For peer review only - http://bmjopen.bmj.com/site/about/guidelines.xhtml

command hallucinations, which was designed to weaken and challenge beliefs about the power of voices, enabling the individual to break free of the need to comply or appease and thereby reduce harmful compliance behaviour and distress.[13,14] This therapeutic model was developed because of a major gap in the evidence base. Although cognitive behaviour therapy is recommended by the UK's National Institute for Health and Care Excellence[15] to reduce overall symptom severity, the guidance notes that there is insufficient evidence for voice compliance and frequency and inconsistent evidence for any direct effect on delusions.

We tested the model in a proof-of-principle trial[16] in a group at high risk of compliance with commands because of recent (<9 months) harmful compliance or appeasement. This operational definition of risk was associated with a 39% rate of recurrence of harmful compliance within 12 months in the control group; in people receiving the therapy, the rate of compliance dropped to 14%, equivalent to an effect size of 1·1. This drop was accompanied by a reduction in the perceived power of the voice; there was a reduction in delusional distress and depression, but this was not maintained at 12 months' follow-up. Crucially, as expected, no change was noted in the frequency or intensity of voices, but only in the (power) relation with them. The results of a similar trial by Shawyer and colleagues[17] of a different therapy based on acceptance of voices by "cultivating the capacity to just notice voices and associated thoughts rather than believe and act on them" showed no effect on compliance or other outcomes. However, this study had a low base rate of compliance.

The aim in the COMMAND trial was to assess the acceptability, effectiveness, and cost-effectiveness of cognitive therapy for command hallucinations. The primary hypothesis was that in participants with command hallucinations who have recently acted on the voices and are therefore at high risk of doing so again cognitive therapy for command hallucinations would increase resistance and thereby reduce the level of further harmful compliance behaviour and the associated risk. Secondary hypotheses were that the perceived power of the persecuting voice would be reduced, which would act as the mediator of change in compliance; there would be no changes in the frequency or topography of voices; and cognitive therapy for command hallucinations would reduce delusional distress and depression.

Methods

Study design

This was a single-blind, prospective, pragmatic randomised controlled trial for the comparison of cognitive therapy for command hallucinations + treatment as usual with treatment as usual alone.[18] Eligible participants were recruited for the trial from three UK centres in Birmingham (which included a site in Leicester), London, and Manchester. Recruitment to the trial began in February, 2008, and was completed in July, 2010. Follow-up assessments began in November, 2008, and were completed in January, 2012. Treatment was for 9 months and follow-up was at 9 months and 18 months after randomisation.

The West Midlands Research Ethics Committee (number 06/MRE07/71) provided ethics approval for the study.

Participants were eligible if they met the following criteria: had International Statistical Classification of Diseases and Related Health Problems, 10th Revision (ICD-10), schizophrenia, schizoaffective (F20, 22, 23, 25, 28, 29), or mood disorders (F32),[19] and were under the care of a clinical team; were aged 16 years and older; had a history of harmful command hallucinations for at least 6 months with recent (<9 months) history of harm to self or others, or major social transgressions as a result of the commands (full or incomplete compliance); or had harmful command hallucinations whereby the individual was distressed and appeasing the powerful voice.

Exclusion criteria were organic impairment or primary diagnosis of addictive disorder or insufficient command of English.

Eligible participants were identified by clinical studies officers from the UK Mental Health Research Network who reviewed electronic case records for history of auditory hallucinations and evidence of risky, aggressive, and violent behaviour. Trained researchers did a screening interview with each participant to confirm eligibility for the trial. After the screening interview, eligible participants were invited to take part and asked to provide informed consent. Once written informed consent had been obtained, the researchers administered a battery of assessments and on completion participants were randomly allocated either to the cognitive therapy for command hallucinations + treatment as usual group or to the treatment as usual only group.

Randomisation and masking

Participants were randomly assigned to the cognitive therapy command hallucinations + treatment as usual group or the treatment as usual only group in a 1:1 ratio using an allocation sequence generated with OpenCDMS.25 and were stratified by the centre with permuted blocks with a randomly varying block size after stratification by centre. OpenCDMS then sent an email notification of the allocation to the therapists and trial manager. After randomisation, an email notification about group allocation was sent to the trial manager, trial administrator, and therapists. An email notification confirming that the participant had been randomly assigned to treatment (with no information about group allocation) was sent to the centre research assistant. The trial administrator then sent a letter to the participant and the care coordinator informing them about the outcome of the randomisation. Thus, the results of the

For peer review only - http://bmjopen.bmj.com/site/about/guidelines.xhtml

randomisation were masked from the assessors and randomisation was independent.

Many strategies were used to assure masked ratings, including research workers who were not involved in the randomisation process; therapists were required to consider potential breaks in masking when planning room use and diary arrangements; and participants were reminded by assessors not to disclose treatment allocation. Overall, 19 (10%) breaks in masking were reported (four in the treatment as usual only group and 15 in the cognitive therapy for command hallucinations + treatment as usual group) of 197 participants. When masking was broken, another rater, masked to group assignment, assessed and rated the participant for all subsequent assessments; accordingly, all final ratings were masked.

Interventions

In cognitive therapy for command hallucinations, cognitive behaviour therapy techniques are used to assess and modify conviction in four beliefs linked to the construct of voice power: the voice has absolute power and control; the individual must comply or appease or be severely punished; the identity of the voice (eg, the Devil); and the meaning attached to the voice (eg, the individual is being punished for a past misdemeanour).

Our protocol for cognitive therapy for command hallucinations was developed by MB and details are provided in our casebook manuals.[13,14] The essence of the therapy is to test the perceived power of the voice by assessment of evidence for the omniscience of the voice, the apparent ability of the voice to predict the future and deliver its threats, and the voice hearer's perceived lack of control over the voice. All trial therapists (qualified clinical psychologists at National Health Service [NHS] band 8a and accredited cognitive therapists) received about 30 h of structured training led by MB and AM. Recordings of cognitive therapy for command hallucinations with pretrial participants were rated according to the cognitive therapy checklist (below). Therapists recording high adherence to cognitive therapy received their first randomly assigned participant. All therapists were highly adherent to cognitive therapy for command hallucinations. Although the intervention is protocol based, differences are recognised in voice content, and beliefs about voices and compliance. Cognitive therapy for command hallucinations differs from previous and generic types and models of cognitive behaviour therapy for psychosis. First, it is informed by a well validated theoretical framework, which can be used to predict an individual's compliance with voices and the associated distress rather than the presence of psychotic symptoms. Second, it adheres to a staged process informed by our cognitive model. Third, the model proposes a single variable that is the target of therapy and also the hypothesised mediator: the power differential between the voice and the hearer of the voice.

The intervention was delivered in NHS clinics with outreach to participants' homes by nine cognitive therapists who were supervised in each centre by a lead clinician with expertise in cognitive behaviour therapy for psychosis. Therapists delivered the intervention only to the cognitive therapy for command hallucinations + treatment as usual group. Group supervision at sites was done once a fortnight with videoconference to monitor adherence to protocol and to minimise centre differences in implementation. Adherence to protocol was monitored with our adapted version of the Cognitive Therapy Checklist[18] using cases selected at random by the trial manager. Acceptability was assessed indirectly by monitoring the rate of treatment completion and dropout; a qualitative method, which will be reported in a further paper, was used.[18] Cognitive therapy for command hallucinations was administered over a maximum of 9 months (about 25 sessions of therapy).

Treatment as usual was provided by community mental health and assertive outreach and early intervention teams. Treatment as usual including antipsychotic medication was documented in accordance with the trial protocol derived from our pilot study.[18]

Outcomes

The compliance or resistance was assessed at baseline (over the previous 9 months), then at 9 months and 18 months after the start of treatment with the Voice Compliance Scale (VCS).[18] This scale is a categorical scale that requires a thorough interview in accordance with the Cognitive Assessment of Voices schedule to obtain a detailed record of all voices and emotional and behavioural responses towards them; and interviewing and using information from, where available, at least one other informant (carers, hostel worker, care coordinator, or psychiatrist). In all cases, care records were scrutinised to search for documented behaviours that were suggestive of compliance. If there was discrepancy between self and informant report that would change a rating, we recorded the more severe level of compliance. This method yields specific behaviours that are assembled into a summary and classified as neither appeasement nor compliance; symbolic appeasement, including mental rehearsal or compliance with innocuous or harmless commands; actual appeasement (ie, preparatory acts or gestures); partial compliance with at least one severe command; and full compliance with at least one severe command. For each of these behaviours, definitions and examples were provided.

29 VCS interviews were selected and rated in a masked fashion by MM for the presence versus absence of full compliance, with an overall κ of 0·73. VCS was assessed for reliability in our pilot trial[16] with similar results to those in this main trial.

The primary outcome was full compliance, assessed after completion of cognitive therapy for command hallucinations (highest score 5).

Secondary outcomes were also assessed. The Voice Power Differential (VPD) Scale[10] was used to measure the

For peer review only - http://bmjopen.bmj.com/site/about/guidelines.xhtml

perceived power differential between voice and voice hearer. Score range was 7–35, with higher scores indicating greater power differential in favour of the voices. This scale has good internal reliability (Cronbach's α=0·85) with high 1 week retest reliability (*r*=0·82). The first item on the scale, the overall power differential between voice and voice hearer, was analysed separately in keeping with our protocol and underlying theory (my voice is much more powerful than me *vs* my voice is much less powerful than me).

The Personal Knowledge Questionnaire and Omniscience Scale[10] was used to measure the hearer's beliefs about the voice's knowledge about personal information (eg, the voice knows everything about me and my past). The score range was 0–15.

The Beliefs About Voices Questionnaire-Revised (BAVQ-R)[20] was used to assess key beliefs about the omnipotence and intentions of the voices, whether benevolent or malevolent, and the emotional and behavioural reactions of the participants towards their voices (resistance *vs* engagement). The scale consists of five subscales: malevolence (score range 0–18), benevolence (0–18), omnipotence (0–18), resistance (0–27), and engagement (0–24). The scale has good test–retest (*r*=0·89) and internal reliability (Cronbach's α=0·85) and is widely used for research into hallucinations.

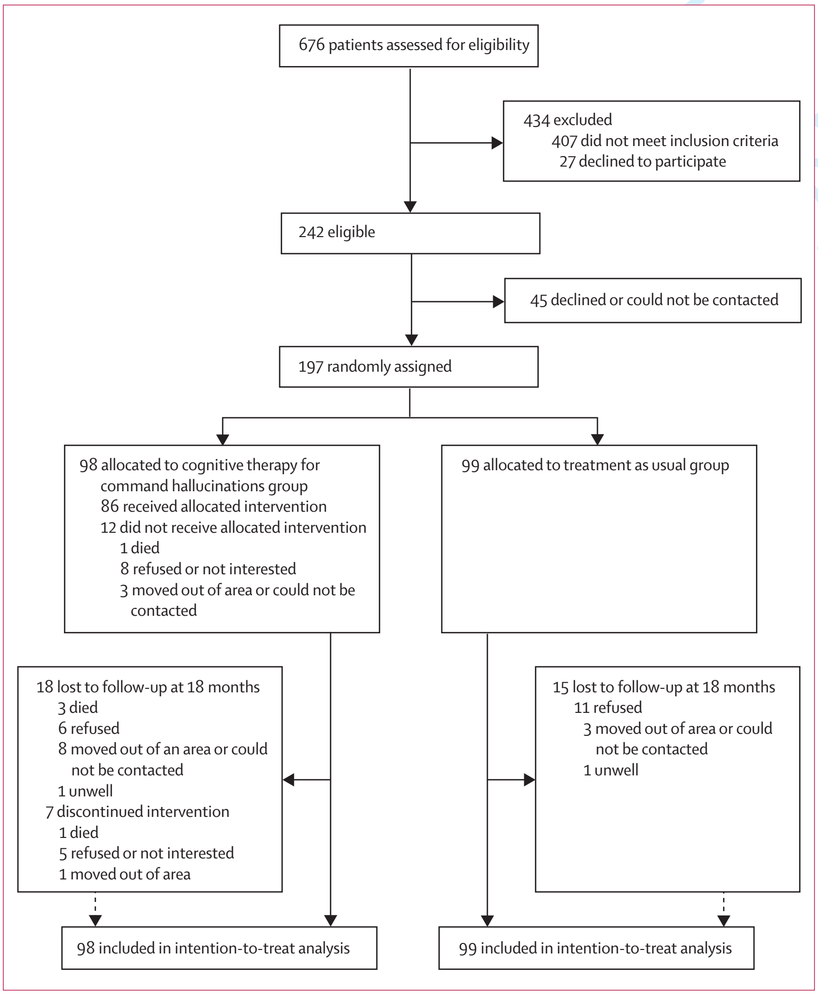

Figure: **Trial profile**

Distress associated with voices was assessed with the Psychotic Symptoms Rating Scales (PSYRATS), Auditory Hallucinations (PSYRATS-AH) subscale.[21] Score range on this subscale was 0–44, with higher scores indicating increasing severity. The items amount of distress and amount of negative content (range 0–4 for both items) are also reported separately. The scale has excellent psychometric properties with good inter-rater reliability for the auditory hallucinations section (range 0·78–1·00).

The Calgary Depression Rating Scale for Schizophrenia[22] is a widely used nine-item observer-rated measure specifically designed for schizophrenia, minimising contamination with negative symptoms and the extrapyramidal side-effects of antipsychotic medication. Score range is 0–27, with higher scores indicating greater severity. The Beck Hopelessness Scale[23] is used to assess feelings about the future, loss of motivation, and expectations; score range is 0–20, with higher scores indicating greater hopelessness. The Beck Scale for Suicidal Ideation allows a thorough assessment of suicidal intent; score range is 0–38, with higher scores indicating an increase in suicidal risk.

The Positive and Negative Syndrome Scale (PANSS)[24] includes scales of positive symptoms (score range 7–49), negative symptoms (7–49), and general psychopathology (16–112); it is used widely in schizophrenia research. The items hallucinations and delusions (1–7 for each item) are also reported separately.

All prescribed antipsychotic medications at baseline and 18 months were recorded and converted to daily dose of olanzapine equivalents, with tables from the International Consensus Study of Antipsychotic Dosing.[25] All adverse events were recorded on standard proforma, at each of the sites, signed off by MB, and collated for the trial data monitoring and ethics committee and the ethics committee.

Statistical analysis

Analysis was by intention to treat. The planned recruitment to the trial was based on a sample size of 100 individuals per group having a greater than 80% power to detect an absolute difference between a proportion who have acted on their voices (VCS score=5) of 40% under treatment as usual group and 20% in the cognitive therapy for command hallucinations group with a Pearson χ^2 test with two-tailed significance of 0·05.

All analyses were done with Stata (version 12). The primary outcome was evaluated with an intention-to-treat analysis with a logistic regression to allow measurement of centre membership and baseline compliance on the VCS. A likelihood-based random effects (intercepts)

For peer review only - http://bmjopen.bmj.com/site/about/guidelines.xhtml

model was used to simultaneously estimate treatment effects at both the 9-month and 18-month follow-up assessments and to allow for missing follow-up data that are assumed to be missing at random. Analogous random-effects models were used for the analysis of the quantitative secondary outcomes. All models included a centre-by-time interaction and a centre-by-baseline-covariate interaction. Any data within a questionnaire scale were prorated with the mean of the remaining items unless there were more than 5% missing data, in which case the scale was designated as missing (this did not apply to VCS)

Because data were from only two follow-up timepoints, no assumptions were needed about the autocorrelation of the longitudinal data. The estimated measures in the initial model included a main effect of treatment and an effect of treatment-by-time interaction; when there was no evidence of an effect of treatment-by-time interaction, the model was simplified by removing the interaction to estimate a treatment effect that was common to both follow-up timepoints.

The trial is registered, number ISRCTN62304114.

Role of the funding source

The design of the trial was peer reviewed by the funders and amended accordingly. The funders had no role in the gathering, analysis, or interpretation of the data, nor in the writing of the paper. MB, GD, and MM had access to the raw data. The corresponding author had full access to all of the data and the final responsibility to submit for publication.

Results

The figure shows the trial profile. 676 participants were screened for eligibility and of these 407 (60%) did not meet the inclusion criteria and 27 (4%) declined to participate. 242 (36%) participants were eligible to participate in the study and 197 (81%) of these were randomly assigned. 45 (19%) individuals declined or could not be contacted after the baseline assessment, before randomisation. Recruitment was completed in July, 2010. The final sample size was 197 participants (64 from Birmingham, 23 Leicester, 62 London, and 48 Manchester), with 98 participants allocated to the cognitive therapy for command hallucinations + treatment as usual group and 99 to the treatment as usual group. 165 (84%) participants were rated as being fully compliant and 32 (16%) as partly compliant or appeasing on VCS at baseline.

164 (83%) of 197 participants completed the 18-month follow-up assessment, 17 (9%) refused to complete the assessment, 11 (6%) could not be contacted, three (2%) died, and two (1%) were unwell. The clinical diagnoses in the sample of 197 participants were schizophrenia (98 [50%]), schizoaffective disorder (29 [15%]), paranoid schizophrenia (17 [9%]), psychosis (50 [25%]), and bipolar disorder (three [2%]). 88 (45%) participants had hospital admissions in the previous 18 months, including 27 (31%) that were compulsory under the UK Mental Health Act. Table 1 shows the different services used by the participants in the 18 months before randomisation. Conversion to olanzapine equivalents was done for 136 (69%) of 197 participants; in 61 (31%) cases, dose was not available or could not be converted. The mean dose of all prescribed antipsychotics, expressed as olanzapine equivalents, was 25·79 mg/day (SD 21·73), including 38 (28%) of 136 participants receiving more than 30 mg/day: 26·88 mg/day (22·02) in the cognitive therapy for command hallucinations + treatment as usual group and 24·77 mg/day (21·55) in the treatment as usual group. At 18 months, the dose was 19·34 mg/day (14·30) in the

	Cognitive therapy for command hallucinations + treatment as usual group (n=98)	Treatment as usual group (n=99)
Inpatient hospital services	53	56
Acute psychiatric ward	45	50
Psychiatric rehabilitation ward	2	3
Long stay psychiatric ward	3	3
Emergency or crisis centre	5	9
General medical ward	14	13
Alcohol or drug treatment ward	0	1
Other	3	5
Hospital outpatient services	82	86
Psychiatric	74	67
Hospital alcohol or substance service	3	4
Non-psychiatric	4	5
Accident and emergency	20	18
Day hospital	4	7
Other	10	15
Community-based services	55	58
Community mental health centre	37	33
Day care centre	18	15
Group therapy	7	3
Sheltered workshop	2	2
Specialist education	3	3
Other	20	24
Primary and community care services	98	98
Family doctor	80	89
Psychiatrist	81	82
Psychologist	13	21
Alcohol or drug treatment or rehabilitation service	14	10
District nurse	3	8
Community psychiatric nurse or case manager	79	80
Social worker	27	26
Occupational therapist	18	21
Voluntary counsellor	1	1
Home help or care worker	15	11
Other	65	63

Data are numbers. *Participants received more than one service.

Table 1: Service use in the 18 months before randomisation*

For peer review only - http://bmjopen.bmj.com/site/about/guidelines.xhtml

	Examples	Compliance	Appeasement
Harm self (n=119)	"Cut yourself"<br>"Drink bleach"<br>"Don't take your medication"	Drinking bleach, cutting or slashing wrists, not taking medication and consequently becoming unwell, or walking in front of cars	Making superficial cuts; mentally rehearsing slashing wrists
Harm others (n=34)	"Attack someone"<br>"Hurt your children"<br>"Smash him or her over the head"	Punching, hitting, and pushing people; hitting children, or beating up partner	Threatening to harm or attack other people; planning how to execute the attack
Kill self (n=42)	"Take an overdose and kill yourself"<br>"Jump off the bridge"<br>"Commit suicide"	Attempted suicide in response to commands by taking an overdose; tied an iron cord around neck; jumping in front of cars	Obtaining tablets and mentally rehearsing taking an overdose; visualising and planning committing suicide by jumping off a bridge
Kill others (n=11)	"Stab your flatmate or sister"<br>"Kill your husband or wife with a knife"<br>"Strangle him"	Attempting to strangle a woman; attacked and grabbed doctor by the throat	Mentally rehearsing killing people; threatening to kill people; buying a knife or axe and planning to kill someone
Destroy property (n=5)	"Burn down the flat"<br>"Smash the windows"<br>"Trash it, destroy it"	Knocked things off the shelves of a shop; hit the door of a car; smashed things in the house	Mentally rehearsing buying petrol and setting fire to the flat
Antisocial behaviour (n=9)	"Start a fight"<br>"Shout at them"	Shouting and swearing at other people; starting arguments with strangers or family	Respond to voices by saying "I will do it later"
Major social transgressions (n=14)	"Steal from a shop"<br>"Rob these people"	Stealing from shops; running naked in the street	Mentally rehearsing and planning how to steal from a shop
Day to day instructions (n=26)	"Eat or don't eat"<br>"Don't wash"	Eat excessively or abstain from eating; not washing; not sleeping	Respond to voices by skipping some meals; appeasement by reassuring the voices that "I will do it later"
Threats (n=11)	"We are out to get you"<br>"We will kill you"	Setting oneself on fire; taking an overdose	Tie a rope around the neck and squeeze hard but then release

Participants might have had more than one command.

Table 2: **Voice commands, compliance, and appeasement behaviour**

cognitive therapy for command hallucinations + treatment as usual group and 19·99 mg/day (14·32) in the treatment as usual group. 115 (58%) of 197 participants received one antipsychotic drug, 61 (31%) received two, and nine (5%) three or more; we were unable to find the medication data for the remaining participants. 32 (16%) of 197 participants were prescribed clozapine at baseline: 16 (50%) in the cognitive therapy for command hallucinations + treatment as usual group and 16 (50%) in the treatment as usual group.

Table 2 shows the nature of the commands heard by participants and the high risk of harmful behaviours associated with the commands. Table 3 shows the baseline characteristics of the whole study sample and the two groups.

Participants allocated to the cognitive therapy for command hallucinations + treatment as usual group received a mean of 19 sessions of cognitive therapy (SD 9, range 0–36), each lasting about 1 h. Adherence to cognitive therapy was excellent, with only 12 (12%) of 98 participants not attending any sessions, and 79 (81%) completing the therapy (all manualised elements). Fidelity to the therapy model was assessed by supervisors EP and AM using the adherence scales with audio recordings. 17 (20%) of 86 participants in the cognitive therapy for command hallucinations + treatment as usual group were randomly selected and 117 of 1500 therapy sessions were rated (8% of all sessions in the cognitive therapy for command hallucinations + treatment as usual group). 72 (62%) of 117 therapy sessions scored higher than the cutoff based on our fidelity protocol (score ≥3 is acceptable use of cognitive therapy for command hallucinations).

55% of the participants in the treatment as usual group complied fully (VCS score 5) with treatment during the first 9 months after randomisation compared with 48% in the cognitive therapy for command hallucinations + treatment as usual group (table 4). In the 9 months after completion of cognitive therapy for command hallucinations + treatment as usual, 46% of the participants in the treatment as usual group complied fully compared with 28% of those in the cognitive therapy for command hallucinations + treatment as usual group (table 4).

The estimated parameters in the initial model included a main effect of treatment and an effect of treatment-by-time interaction. The treatment-by-time interaction indicates how the rates of compliance in the two treatment groups diverge over time. At 9 months, the odds ratio was 0·74 (95% CI 0·40–1·39, p=0·353). At 18 months, the odds ratio was 0·45 (0·23–0·88, p=0·021). Although both odds ratios are less than 1, there might be a discrepancy between the treatment effects at the two follow-up timepoints. However, the treatment-by-time interaction (a measure of this discrepancy), with an estimated odds ratio of 0·54 (0·27–1·1, p=0·091), was not significant and was therefore removed from the model. The resulting estimate of the treatment effect, interpreted as the effect that is common to both follow-up timepoints, was an odds ratio of 0·57 (0·33–0·98, p=0·042).

For peer review only - http://bmjopen.bmj.com/site/about/guidelines.xhtml

	Whole sample (n=197)	Cognitive therapy for command hallucinations + treatment as usual group (n=98)	Treatment as usual group (n=99)
Age (years)	37·4 (12·1; 16–64)	38·8 (12·2; 16–63)	35·9 (11·9; 17–64)
Age at onset (years)	22·14 (10·56; 5–63), n=178	21·80 (10·27; 5–57)	22·48 (10·89; 11–63)
Sex, male:female	113:84	61:37	52:47
VPD total	26·95 (5·05; 9–35), n=191	26·54 (5·45; 9–35), n=95	27·36 (4·62; 13–35), n=96
VPD power	3·92 (1·10; 1–5), n=195	3·87 (1·18; 1–5), n=97	3·98 (1·03; 1–5), n=98
PANSS positive	19·38 (4·86; 10–33), n=197	19·11 (4·94; 11–32), n=98	19·64 (4·79; 10–33), n=99
PANSS hallucinations	5·28 (1·12; 2–7), n=197	5·21 (1·18; 2–7), n=98	5·34 (1·05; 2–7), n=99
PANSS delusions	3·73 (1·50; 1–7), n=197	3·69 (1·67; 1–7), n=98	3·77 (1·33; 1–7), n=99
PANSS negative	16·02 (6·29; 7–34), n=195	15·86 (6·30; 7–33), n=97	16·18 (6·31; 7–34), n=98
PANSS general	36·37 (8·60; 17–71), n=195	35·78 (8·83; 17–61), n=97	36·96 (8·38; 20–71), n=98
PANSS total	71·73 (16·56; 38–132), n=195	70·74 (17·11; 38–121), n=97	72·71 (16·02; 39–132), n=98
PSYRATS hallucinations (total)	32·63 (4·40; 18–41),n=194	32·27 (4·49; 18–41), n=95	32·97 (4·31; 21–41), n=99
PSYRATS distress (amount)	3·22 (0·85; 0–4), n=196	3·22 (0·83; 1–4), n=97	3·22 (0·87; 0–4), n=99
PSYRATS frequency	2·94 (1·21; 0–4), n=197	2·98 (1·22; 0–4), n=98	2·91 (1·21; 0–4), n=99
PSYRATS negative content (amount)	3·37 (0·78; 0–4), n=196	3·41 (0·81; 0–4), n=97	3·32 (0·74; 0–4), n=99
Calgary Depression	12·09 (6·01; 0–27), n=197	12·44 (6·33; 0–27), n=98	11·75 (5·68; 0–26), n=99
Beck Hopelessness	10·72 (5·39; 1–20), n=193	11·05 (5·33; 1–19), n=96	10·39 (5·46; 1–20), n=97
Beck Suicide Ideation	9·91 (9·37; 0–34), n=196	10·59 (9·64; 0–34), n=97	9·24 (9·10; 0–28), n=99
Personal Knowledge Questionnaire	10·99 (3·22; 1–15), n= 197	10·29 (3·32; 2–15), n=98	10·90 (3·10; 1–15), n=99
BAVQ-R malevolence	13·05 (4·33; 2–18), n=197	12·93 (4·46; 2–18), n=98	13·17 (4·22; 2–18), n=99
BAVQ-R benevolence	3·37 (3·99; 0–15), n=196	3·22 (3·82; 0–14), n=97	3·52 (4·16; 0–15), n=99
BAVQ-R omnipotence	13·49 (3·75; 0–18), n= 197	13·23 (3·77; 0–18), n=98	13·74 (3·72; 3–18), n=99
BAVQ-R total resistance	21·38 (4·86; 5–27), n=197	21·29 (5·12; 5–27), n=98	21·47 (4·60; 10–27), n=99
BAVQ-R emotional resistance	9·85 (2·30; 0–12), n=197	9·48 (2·48; 1–12), n=98	10·22 (2·06; 4–12), n=99
BAVQ-R behavioural resistance	11·53 (3·60; 0–15), n=197	11·81 (3·63; 2–15), n=98	11·25 (3·57; 0–15), n=99
BAVQ-R total engagement	5·06 (4·92; 0–21), n=197	4·87 (4·58; 0–19), n=98	5·25 (5·25; 0–21), n=99
BAVQ-R emotional engagement	1·99 (2·84; 0–12), n=197	1·82 (2·57; 0–12), n=98	2·16 (3·08; 0–11), n=99
BAVQ-R behavioural engagement	3·07 (2·97; 0–12), n=197	3·05 (2·84; 0–11), n=98	3·09 (2·92; 0–12), n=99

Data are mean (SD; range), unless otherwise indicated. Some scales have missing data and numbers are provided to indicate sample with complete data. VPD=Voice Power Differential. PANSS=Positive and Negative Syndrome Scale. PSYRATS=Psychotic Symptoms Rating Scales. BAVQ-R=Beliefs About Voices Questionnaire-Revised.

Table 3: **Baseline characteristics of participants**

The independent data monitoring and ethics committee monitored all serious adverse events; the committee found no serious adverse events attributable to the therapy.

As with compliance, the treatment-by-time interaction was not significant and we therefore report the effect of treatment for the two follow-up timepoints. For the VPD total, the estimated treatment effect (adjusted mean outcome for the cognitive therapy for command hallucinations + treatment as usual group minus the corresponding mean for the treatment as usual group) common to both timepoints was –1·82 (95% CI –3·46 to –0·18, p=0·03). For the VPD power differential item, the estimated treatment effect for both timepoints was –0·52 (–0·849 to –0·185, p=0·002).

Treatment effects for all other outcomes (PSYRATS total and distress, Calgary Depression, Beck Hopelessness and Beck Suicidal Ideation, BAVQ-R, and knowledge [omniscience] scale) were not significant (appendix). Notably, all secondary outcomes fell significantly over time, equally in both treatment groups (table 3; table 4).

No differences were noted between groups at baseline or follow-up in the total amount of antipsychotics prescribed (olanzapine equivalents). 32 (16%) of 197 participants received cognitive behaviour therapy as part of their routine care throughout the trial: 17 (53%) of 32 in the treatment as usual group and 15 (47%) in the cognitive therapy for command hallucinations + treatment as usual group (not concurrently with cognitive therapy for command hallucinations). There was no difference between the groups in baseline or follow-up PANSS total or subscale scores (table3; table 4).

Discussion

By 18 months' follow-up, 46% of the participants in the treatment as usual group had at least one episode of full compliance compared with 28% in the cognitive therapy for command hallucinations + treatment as usual group (table 4). Hence, the cognitive therapy for command hallucinations + treatment as usual group

See Online for appendix

For peer review only - http://bmjopen.bmj.com/site/about/guidelines.xhtml

	9 months		18 months	
	Cognitive therapy for command hallucinations + treatment as usual group (n=98)	Treatment as usual group (n=99)	Cognitive therapy for command hallucinations + treatment as usual group (n=98)	Treatment as usual group (n=99)
Full compliance	41/85 (48%)	49/89 (55%)	22/79 (28%)	39/85 (46%)
VPD total	21·31 (5·86; 7–33), n=87	23·98 (6·41; 11–35), n=85	22·39 (6·21; 9–34), n=75	23·42 (6·88; 7–35), n=81
VPD power	2·80 (1·18; 1–5), n=87	3·34 (1·36; 1–5), n=86	2·82 (1·29; 1–5), n=76	3·20 (1·42; 1–5), n=81
PSYRATS total	29·10 (7·57; 0–42), n=82	28·05 (8·55; 0–40), n=84	28·63 (5·93; 7–38), n=75	28·00 (8·88; 0–41), n=76
PSYRATS distress	2·83 (1·26; 0–4), n=82	2·52 (1·49; 0–4), n=84	2·6 (1·24; 0–4), n=75	2·70 (1·39; 0–4), n=76
PSYRATS frequency	2·49 (1·44; 0–4), n=85	2·42 (1·5; 0–4), n=89	2·39 (1·32; 0–4), n=76	2·23 (1·59; 0–4), n=82
PSYRATS negative content (amount)	3·06 (1·17; 0–4), n=83	2·88 (1·30; 0–4), n=85	3·09 (1·11; 0–4), n=75	3·10 (1·18; 0–4), n=77
PANSS positive	16·06 (4·53; 7–27), n=86	17·85 (5·51; 7–35), n=88	16·96 (5·32; 7–32), n=78	17·30(5·78; 7–35), n=83
PANSS negative	12·94 (5·22; 7–33), n=86	13·45 (4·97; 7–26), n=88	13·33 (5·47; 7–31), n=78	12·96 (4·48; 7–25), n=83
PANSS hallucinations	4·36 (1·71; 1–7), n=86	4·69 (1·59; 1–7), n=88	4·42 (1·55; 1–7), n=78	4·28 (1·62; 1–7), n=83
PANSS delusions	3·13 (1·50; 1–6), n=86	3·43 (1·42; 1–7), n=88	3·18 (1·49; 1–7), n=78	3·28 (1·60; 1–6), n=83
PANSS general	30·85 (8·36; 16–59), n=86	32·64 (9·10; 16–57), n=88	31·22 (8·40; 16–56), n=78	32·73 (9·36; 17–57), n=83
PANSS total	59·85 (14·24; 30–98), n=86	63·94 (16·26; 33–111), n=88	61·51 (15·46; 31–101), n=78	63·00 (16·92; 32–107), n=83
Calgary depression scale	8·77 (6·04; 0–24), n=86	8·76 (6·24; 0–25), n=87	7·79 (6·33; 0–23), n=78	7·36 (4·97; 0–20), n=83
Beck hopelessness scale	8·17 (5·01; 1–19), n=81	8·72 (5·62; 1–19), n=83	8·77 (5·09; 1–19), n=77	8·31 (5·58; 1–20), n=80
Beck Scale for Suicide Ideation	6·05 (9·11; 0–34), n=85	5·88 (8·74; 0–27), n=86	4·35 (6·98; 0–28), n=77	5·30 (8·22; 0–27), n=79
Personal Knowledge Questionnaire	10·15 (3·40; 0–15), n= 86	9·58 (3·74; 0–15), n= 86	10·51 (3·41; 0–15), n=75	10·38 (3·98; 0–15), n=79
BAVQ–R malevolence	10·60 (5·15; 0–18), n=87	11·41 (4·93; 0–18), n=86	11·05 (5·38; 0–18), n=75	11·65 (5·22; 0–18), n=79
BAVQ–R benevolence	3·18 (4·52; 0–17), n=87	3·28 (4·47; 0–18), n=86	2·77 (3·75; 0–13), n=74	2·86 (4·06; 0–15), n=79
BAVQ–R omnipotence	10·29 (4·46; 0–18), n=87	11·38 (4·85; 0–18), n=86	10·03 (5·07; 0–18), n=74	11·23 (4·82; 0–18), n=79
BAVQ–R total resistance	20·41 (5·99; 0–27), n=86	19·95 (6·09; 2–27), n=86	20·27 (5·61; 2–27), n=73	20·95 (5·85; 2–27), n=78
BAVQ–R emotional resistance	8·77 (2·95; 0–12), n=86	8·83 (3·09; 0–12), n=86	8·49 (3·38; 0–12), n=73	9·08 (3·30; 0–12), n=79
BAVQ–R behavioural resistance	11·64 (3·87; 0–15), n=86	11·13 (3·97; 0–15), n=86	11·78 (3·72; 0–15), n=73	11·90 (3·64; 0–15), n=78
BAVQ–R total engagement	4·27 (5·20; 0–23), n=86	4·70 (5·28; 0–22), n=86	4·05 (5·60; 0–17), n=73	4·23 (5·24; 0–24), n=78
BAVQ–R emotional engagement	1·80 (3·12; 0–12), n=86	2·27 (3·15; 0–12), n=86	1·70 (2·61; 0–11), n=73	2·01 (3·27; 0–12), n=79
BAVQ–R behavioural engagement	2·47 (2·82; 0–11), n=86	2·43 (2·66; 0–11), n=86	2·36 (2·91; 0–11), n=73	2·19 (2·87; 0–12), n=78

Data are n/N (%) or mean (SD; range), unless otherwise indicated. Higher scores indicate poorer outcome. Some scales have missing data and numbers are provided to indicate sample with complete data. VPD=Voice Power Differential. PSYRATS=Psychotic Symptoms Rating Scales. PANSS=Positive and Negative Syndrome Scale. BAVQ-R=Beliefs About Voices Questionnaire-Revised.

Table 4: **Primary and secondary outcomes at 9 months and 18 months**

had a large, significant, and clinically meaningful reduction in the rate of compliance compared with the treatment as usual group (odds ratio 0·45). The odds ratio of the combined treatment effect at both follow-up timepoints was 0·57. This effect was matched by a change in the singular focus of the intervention: the voices' perceived power to deliver a supposed threat to the individual (strictly the power difference between the voices' ability to deliver the threat and the voice hearer's ability to withstand and mitigate the threat). In accord with our model, changes in power might have mediated change in compliance. This outcome requires a complex analysis, which we will present in future papers alongside the results of a health-economic analysis. We believe that the results of this trial represent an important advance in the treatment of individuals with a high risk of committing harm to themselves or others. Reducing risk in those who have previously acted on their psychotic symptoms has not yet proved possible, other than ensuring that the best evidence-based treatment is provided and, as a last resort, the use of compulsory detention, which has been steadily rising in the UK and across Europe.[26]

In accord with the findings from our pilot study,[16] no treatment effect was noted for psychotic symptoms, including hallucinations. Unlike the pilot study, however, there was no effect of cognitive therapy for command hallucinations on distress linked to voices or on depression; in the pilot study, these changes were lost at follow-up and the results of this trial confirm absence of effect. Importantly, most of the symptoms measured in the current study fell significantly over time in both groups (as in the pilot study), probably indicating that the sample was identified at baseline at the peak of compliance and distress. In the treatment as usual group, despite improvement in symptoms and voice frequency with time, participants showed a much higher rate of compliance, with a fairly unchanged appraisal of the voices' power, emphasising the importance of this appraisal. The effect of cognitive

For peer review only - http://bmjopen.bmj.com/site/about/guidelines.xhtml

therapy for command hallucinations seemed greater at 18 months than at 9 months; however, we should be cautious in coming to this conclusion because the analyses (testing for the time-by-treatment interaction) showed that the difference in the treatment effects at 9 months and 18 months was not significant. Nevertheless, the effect at 9 months was not significant, suggesting that cognitive therapy for command hallucinations might have had a delayed effect. This explanation is plausible because the intervention was delivered slowly and steadily over 9 months to vulnerable participants; thus, participants might have been more compliant during this period, before the intervention had time to fully take effect.

The results of the COMMAND trial confirm the size and focal nature of the effect of cognitive therapy for command hallucinations on power and compliance, first reported in our pilot trial. Cognitive therapy for command hallucinations was acceptable to clients, as shown by the high rate (>80%) of completion of treatment, among the highest reported for cognitive behaviour therapy (or indeed other treatment) for psychosis. Cognitive therapy for command hallucinations was not designed to be a panacea for all difficulties linked to psychosis and commanding voices, but to target this one key appraisal (power differential) and behaviour (full compliance) for which it appears to be successful. At the end of therapy, many participants remained significantly depressed and continued to be distressed by their voices. Other interventions will be needed to address these (eg, conventional cognitive behaviour therapy for psychosis). Our recent longitudinal study of voice compliance[27] showed that in addition to voice power, impulsivity, but not anger, was a predictor, suggesting other potential intervention targets to improve outcome with cognitive therapy for command hallucinations (panel).

So far, COMMAND is the largest trial of treatment for the high-risk group of individuals who are traditionally difficult to study and to follow up; it is also one of the few trials focusing on risk of serious harm in psychosis, and the only such trial with commanding hallucinations. The high rate of consent to randomisation and level of completed follow-up (both >80%) assures the validity and generalisability of the findings (the high rate of consent probably indicates treatment resistance and harm linked to this symptom). The primary outcome, the presence of any episode of full compliance in the follow-up, was a stringent test of the hypothesis. In situations in which the frequency of full compliance might have fallen, the rating was given as full compliance, potentially underestimating the treatment effect; for example, there were many instances in which major episodes of self-harm fell sharply (eg, from weekly to once in 9 months) but this benefit was not indicated in the outcome. This limitation will need to be addressed in future work. Adherence to treatment was excellent; however, the rated level of fidelity to cognitive therapy for command hallucinations, based on a random set of recordings, was good (62%), but not as high as expected. There were two broad reasons for this limitation. First, therapists strayed from pure cognitive therapy for command hallucinations to include broader cognitive behaviour therapy, probably because of the complexity of the clients. Second, clients were often distressed by the voices within sessions, requiring therapists to spend much time encouraging communication and talking about neutral topics, which could not be rated like cognitive therapy for command

***Panel:* Research in context**

Systematic review

Two systematic reviews of cognitive behaviour therapy for psychosis have been reported.[28,29] The results of the first systematic review[28] showed small to moderate effect sizes of therapy on positive psychosis symptoms. The results of the second,[29] a Cochrane review, of the comparison of cognitive behaviour therapy with a control psychosocial intervention, showed that definitive conclusions could not be drawn because of an insufficient number of trials, but there was some evidence of equivalence. The results of a rigorous trial in *The Lancet*[30] showed cognitive behaviour therapy to be effective in participants who had refused medication (all previous trials were of participants who were on medication). The results of a systematic review,[28] which included our pilot trial[16] with the largest effect size, showed substantial heterogeneity, indicating that cognitive behaviour therapy might have better effectiveness in some contexts. The recent updated guidance from the National Institute for Health and Care Excellence for schizophrenia[15] lends support to this view.

Interpretation

To the best of our knowledge, COMMAND is the first large-scale trial to test the effect of cognitive behaviour therapy on harmful behaviour, rather than overall psychosis symptoms. The results are strong evidence of substantial reductions in harmful compliance. This outcome must be viewed in the context of the high-risk criteria we used. According to our results, individuals showing evidence of recent harmful compliance (<9 months) are at high risk of further compliance within 18 months (46%). Whether individuals who hear commands, but have yet to comply or do so infrequently, can be prevented from future compliance is not known. Because the cognitive behaviour therapy we used seems to be highly acceptable and most individuals completed the treatment, it might have wider application in some troublesome auditory hallucinations. Results of COMMAND resolve the uncertainty about the effectiveness of cognitive behaviour therapy in reducing harm from commanding hallucinations, and suggest that the next generation of trials will be more effective if they are theory driven and target behaviour or distress, rather than psychosis symptoms.

For peer review only - http://bmjopen.bmj.com/site/about/guidelines.xhtml

hallucinations. These fidelity issues will be addressed in further papers drawing on the qualitative aspect of the trial protocol. The possibility of therapist effects needs to be acknowledged; the contribution of non-protocol-based therapeutic effects might create clustering within the data and therefore lead to imprecision in the actual effects. However, the benefits of cognitive therapy for command hallucinations might be due to qualities of some therapists that are not captured in the manualised treatment, which may weaken the effect of cognitive therapy for command hallucinations in routine care.

Cognitive behaviour therapy for psychosis has become a complex package and recent meta-analyses have shown that its effect is smaller than suggested by the results of initial trials. Trials of the comparison of cognitive behaviour therapy with treatment as usual might have limitations due to poor treatment as usual, as reported by the Schizophrenia Commission; however, as we report here, treatment as usual was characterised by a very high level of treatment and service contact with the participants due to the concern about the risks. The absence of an active control intervention raises the possibility that a non-specific aspect of cognitive therapy for command hallucinations (eg, extra treatment time) might account for some or all of the effect size. If this were the case, we would have expected non-specific changes in many outcome domains; however, as predicted, we noted changes in perceived voice power and compliance, but not psychosis or other symptoms. Another, equally credible therapy might have achieved the same outcome. We decided first to establish whether this theoretically informed intervention could have a clinically significant effect on treatment-refractory and high-risk participants. The results of the COMMAND trial now open the way for more complex and expensive trials to resolve these questions. We have previously argued,[31] however, that the next generation of trials of cognitive behaviour therapy should move away from the conventional goal of reducing psychotic symptoms in participants to focus on the core goal of cognitive behaviour therapy—ie, changing affect and behaviour—with theoretically informed and more focused interventions. In this respect COMMAND might be considered a model of the next generation of cognitive behaviour therapy trials for psychosis.

For the **report by the Schizophrenia Commission** see http://www.schizophreniacommission.org.uk

Contributors

MB, EP, SL, TW, NT, LD, and GD designed the study. MB, MM, AM, EP, TW, NT, and SL administered the study. MM was the trial manager. MB developed the cognitive therapy for command hallucinations. MB and AM designed and led the training for cognitive therapy for command hallucinations, and AM and EP led the supervision process and the rating of therapy tapes. GD designed and undertook the statistical analyses and LD designed and undertook the economic analyses. All authors contributed to the interpretation of the data, the writing of the paper, and approved the final manuscript. MB had full access to all the data in the study and had final responsibility for the decision to submit for publication.

Declaration of interests

We declare that we have no competing interests.

Acknowledgments

The trial was funded by the UK Medical Research Council (G0500965). We acknowledge the support of the National Institute for Health Research (NIHR) Mental Health Research Network in recruiting participants; the trial steering committee (chair Elizabeth Kuipers) and the data monitoring and ethics committee (chair Andrew Gumley), for their assistance in setting up, conducting, and monitoring the trial. Helen Lester who was a co-investigator on COMMAND, sadly died in March, 2013. We thank the clinical teams and service users for their continuing support and participation in the trial. We would also particularly like to acknowledge the work of the researchers and therapists involved in the trial. MB was part funded by the NIHR through the Collaborations for Leadership in Applied Health Research and Care for the West Midlands. EP, NT, and TW acknowledge support from the NIHR Biomedical Research Centre for Mental Health at King's Health Partners, London, UK. The views expressed in this publication are not necessarily those of the NIHR or the Department of Health management group.

References

1 Van Os J, Kapur S. Schizophrenia. *Lancet* 2009; **374:** 635–45.
2 Fazel S, Gulati G, Linsell L, Geddes JR, Grann M. Schizophrenia and violence: systematic review and meta-analysis. *PLoS Med* 2009; **6:** 1–14.
3 Department of Health. No health without mental health, a cross-government mental health outcomes strategy for people of all ages. UK: Department of Health, 2011.
4 Leucht S, Arbter D, Engel RR, Kissling W, Davis JM. How effective are second generation antipsychotic drugs? A meta-analysis of placebo-controlled trials. *Mol Psychiatry* 2009; **14:** 429–47.
5 Nose M, Barbui C, Tansella M. How often do patients with psychosis fail to adhere to treatment programmes? A systematic review. *Psychol Med* 2003, **33:** 1149–60.
6 Shawyer F, Mackinnon A, Farhall J. Command hallucinations and violence: implications for detention and treatment. *Psychiatry, Psychol Law* 2003, **10:** 97–107.
7 Rogers R, Watt A, Gray NS, MacCulloch M, Gournay K. Content of command hallucinations predicts self harm but not violence in a medium secure unit. *J Forens Psychiatry* 2002; **13:** 251–62.
8 Appelbaum PS, Robbins P, Monahan J. Violence and delusions: data from the MacArthur Violence Risk Assessment study. *Am J Psychiatry* 2000; **157:** 566–72.
9 Chadwick P, Birchwood M. The omnipotence of voices: a cognitive approach to auditory hallucinations. *B J Psychiatry* 1994; **164:** 190–201.
10 Birchwood M, Gilbert P, Gilbert J, et al. Do interpersonal schema influence the relationship with the dominant 'voice' in schizophrenia? A comparison of three models. *Psychol Med* 2004; **34:** 1571–80.
11 Connor C, Birchwood M. Power and perceived expressed emotion of voices: their impact on depression and suicidal thinking in those that hear voices. *Clin Psychol Psychother* 2013; **20:** 199–205.
12 Fox J, Gray N, Lewis N. Factors determining compliance with violent content: the role of social rank, perceived power of the voice and voice malevolence. *J Forens Psychiatry Psychol* 2004, **15:** 511–31.
13 Byrne S, Birchwood M, Trower P, Meaden A. A Casebook of cognitive behaviour therapy for command hallucinations. A social rank theory approach. London and New York: Routledge, 2006.
14 Meaden A, Keen N, Aston R, Barton K, Bucci S. Cognitive therapy for command hallucinations: an advanced practical companion. Hove: Routledge, 2013.
15 NICE. Psychosis and schizophrenia in adults. London: National Institute for Health and Care Excellence, 2014.
16 Trower P, Birchwood M, Meaden A, Byrne S, Nelson A, Ross K. Cognitive therapy for command hallucinations: randomised controlled trial. *Br J Psychiatry* 2004; **184:** 312–20.
17 Shawyer F, Farhall J, Mackinnon A, et al. A randomised controlled trial of acceptance-based cognitive behavioural therapy for command hallucinations in psychotic disorders. *Behav Res Ther* 2012; **50:** 110–21.
18 Birchwood M, Peters E, Tarrier N, et al. A multi-centre, randomised controlled trial of cognitive therapy to prevent harmful compliance with command hallucinations. *BMC Psychiatry* 2011; **11:** 155–58.

For peer review only - http://bmjopen.bmj.com/site/about/guidelines.xhtml

19 WHO. The ICD-10 Classification of Mental and Behavioural Disorders. Geneva: World Health Organization, 2009.

20 Chadwick P, Lees S, Birchwood M. The revised beliefs about voices questionnaire (BAVQ-R). *Br J Psychiatry* 2000; **177:** 229–32.

21 Haddock G, McCarron J, Tarrier N, Faragher EB. Scales to measure dimensions of hallucinations and delusions: the psychotic symptom rating scales (PSYRATS). *Psychol Med* 1999; **29:** 879–89.

22 Addington D, Addington J, Maticka-Tyndale E. Assessing depression in schizophrenia: the Calgary Depression Scale. *Br J Psychiatry* 1993; **22:** 39–44.

23 Beck AT. The Beck Hopelessness Scale. Alexandria: VA: Psychological Corporation, 1978.

24 Kay SR, Opler LA, Fiszbein A. Positive and Negative Syndrome Scale (PANSS) rating manual. San Rafael, CA: Social and Behavioural Sciences Documents, 1987.

25 Gardner DM, Murphy AL, O'Donnell, H. International consensus study of antipsychotic dosing. *Am J Psychiatry* 2010; **167:** 686–93.

26 Salize HJ, Dressing H. Epidemiology of involuntary placement of mentally ill people across the European Union. *Br J Psychiatry* 2004; **184:** 163–68.

27 Bucci S, Birchwood M, Twist L, Tarrier N, Emsley R, Haddock G. Predicting compliance with command hallucinations: anger, impulsivity and appraisals of voices' power and intent. *Schizophr Res* 2013; **147:** 163–68.

28 Jauhar S, McKenna PJ, Radua J, Fung E, Salvador R, Laws KR. Cognitive-behavioural therapy for the symptoms of schizophrenia: systematic review and meta-analysis with examination of potential bias. *Br J Psychiatry* 2014; **204:** 20–29.

29 Jones C, Hacker D, Cormac I, Meaden A, Irving CB. Cognitive behaviour therapy versus other psychosocial treatments for schizophrenia. *Cochrane Database Syst Rev* 2012; **4:** CD008712.

30 Morrison AP, Turkington D, Pyle M, et al. Cognitive therapy for people with schizophrenia spectrum disorders not taking antipsychotic drugs: a single-blind randomised controlled trial. *Lancet* 2014; **383:** 1395–403.

31 Birchwood M, Trower P. The future of cognitive-behavioural therapy for psychosis: not a quasi-neuroleptic. *Br J Psychiatry* 2006; **188:** 107–08.

For peer review only

For peer review only - http://bmjopen.bmj.com/site/about/guidelines.xhtml

COREQ Criteria checklist

No. Item	Guide questions/description	Reported on Page #
Domain 1: Research team and reflexivity		
**Personal Characteristics**		
1. Interviewer/facilitator	Individual interviews were conducted HL (co-applicant) and research assistants (KP, LB)	Page 4
2. Credentials	HL: MD, PhD<br>KP: BSc Psychology<br>LB:BSc in Psychology, MSc in Forensic mental Health science	Page 4
3. Occupation	HL: Professor of Primary Care<br>KP, LB: research assistants	Page 4
4. Gender	All female	Page 4
5. Experience and training	All interviewers have been trained in qualitative research and have conducted qualitative interviews and focus groups in the past.	Page 4
Relationship with participants		
6. Relationship established	HL was unknown to participants prior to study commencement. KP and LB were known to participants (during baseline and follow up data collection).	Page 4
7. Participant knowledge of the interviewer	At the beginning of the interview the participants were informed of the interviewers' background and expertise.	Page 4
8. Interviewer characteristics	The participants understood the purpose of the interview to look into their thoughts and experiences of CTCH, which they had recently received. They understood that their views about their therapist would not be discussed with therapist.	Page 4
Domain 2: study design		

For peer review only - http://bmjopen.bmj.com/site/about/guidelines.xhtml

Theoretical framework		
9. Methodological orientation and Theory	Grounded theory	Page 5
**Participant selection**		
10. Sampling	A sample of 25 participants was drawn, who were selected randomly by number generator, from the total of 98 receiving the therapy in the trial (25.5%).	
11. Method of approach	Via telephone	
12. Sample size	25 participants	
13. Non-participation	Those who did not complete the course of therapy	
**Setting**		
14. Setting of data collection	In environments convenient to the participant and the interviewer, usually a room at the centre the therapy was delivered but also could be participants own home.	Page 4
15. Presence of non-participants	A few participants had a friend/relative in the room with them at the participants' request. This was permitted.	Page 4
16. Description of sample	The sample consisted of 25 participants, 15 of whom were males. The mean age of the sample was 42 years, and 68% were British. Thirty-six per cent (36%) of the sample was diagnosed as 'unspecified psychosis', 56% as 'Schizophrenia' and 8% as Schizo-affective disorder.	Page 4
**Data collection**		
17. Interview guide	Interviews used a semi structured approach, with a list of questions to ask and subsidiary questions related to participant responses.	Page 4
18. Repeat interviews	No repeat interviews were conducted.	Page 4

For peer review only

19. Audio/visual recording	Audio recording, from which interviews were transcribed verbatim	Page 4
20. Field notes	Notes were kept during the interviews in the form of memos.	Page 4
21. Duration	Each interview lasted approximately 30 minutes.	Page 4
22. Data saturation	We believed data saturation to have been achieved by our sample of 25 participants.	Page 4
23. Transcripts returned	Transcripts were not returned to participants	Page 4
Domain 3: analysis and findings		
**Data analysis**		
24. Number of data coders	Two coders LM and EE	Page 5
25. Description of the coding tree	No	
26. Derivation of themes	Themes were derived from the data	Page 6
27. Software	None used	Page 5
28. Participant checking	None used.	Page 5
**Reporting**		
29. Quotations presented	yes	Page 6 onwards
30. Data and findings consistent	Yes and presented in context of literature	Page 6 onwards
31. Clarity of major themes	2 main themes	Page 6 onwards
32. Clarity of minor themes	11 minor themes	Page 6 onwards

For peer review only

BMJ Open

The COMMAND trial of cognitive therapy for harmful compliance with command hallucinations (CTCH): a qualitative study of acceptability and tolerability in the UK.

Journal:	*BMJ Open*
Manuscript ID	bmjopen-2018-021657.R1
Article Type:	Research
Date Submitted by the Author:	03-May-2018
Complete List of Authors:	Birchwood, Max; University of Warwick, Division of mental health & well-being<br>Mohan, Laura; University of Birmingham, College of Medicine<br>Meaden, Alan; Birmingham and Solihull Mental Health NHS Foundation Trust<br>Tarrier, Nick; University of Manchester<br>Lewis, Shon; University of Manchester<br>Wykes, Til; King's College London (Institute of Psychiatry),<br>Davies, Linda; University of Manchester, Institute of Population Health<br>Dunn, Graham; University of Manchester, Health Methodology Research<br>Peters, Emmanuelle; Kings College, London, IPPN<br>Michail, Maria; University of Birmingham, School of Psychology
**Primary Subject Heading**:	Mental health
Secondary Subject Heading:	Qualitative research, Mental health
Keywords:	Command hallucinations, Psychosis, CBT, Harm

SCHOLARONE™
Manuscripts

The COMMAND trial of cognitive therapy for harmful compliance with command hallucinations (CTCH): a qualitative study of acceptability and tolerability in the UK.

Max Birchwood*, Laura Mohan, Alan Meaden, Nick Tarrier, Shon Lewis, Til Wykes, Linda Davies, Graham Dunn, Emmanuelle Peters and Maria Michail

Warwick Medical School, Mental Health and Wellbeing, University of Warwick, UK (Prof M Birchwood DSc)

College of Medicine and Dentistry, University of Birmingham (L Mohan),

Faculty of Health Science, Birmingham City University, Birmingham, UK (Professor A Meaden, D Clin Psy);

Division of Psychology & Mental Health (Prof Lewis, PhD and Prof Tarrier, PhD.)

School of Psychology, Institute for Mental Health, University of Birmingham (M Michail, PhD)

Centre for Biostatistics, Institute of Population Health, University of Manchester, Manchester, UK (Prof G Dunn PhD)

Kings College, Institute of Psychiatry University of London (E Peters PhD, Prof T Wykes, PhD)

* Corresponding author: Professor Birchwood, m.j.birchwood@warwick.ac.uk

For peer review only - http://bmjopen.bmj.com/site/about/guidelines.xhtml

For peer review only

Abstract

Objectives: To explore service user experiences of a 9-month cognitive behavioural therapy for command hallucinations in the context of a randomized controlled trial including their views on acceptability and tolerability of the intervention.

Design: Qualitative study using semi-structured interviews.

Setting: The study took place across three sites: Birmingham, Manchester and London. Interviews were carried out at the sites where therapy took place, which included service bases and participants' homes.

Participants: Of 197 patients who consented to the trial, 98 received the CTCH intervention; 25 (15 males) of whom were randomly selected and consented to the qualitative study. The mean age of the sample was 42 years, and 68% were White British.

Results: Two superordinate themes were identified: participants' views about the aspects of CTCH they found most helpful; and, participants' concerns with therapy. Helpful aspects of the therapy included: gaining control over the voices; challenging the power and omniscience of the voices; following a structured approach; normalisation and mainstreaming of the experience of voices; and having peer support alongside the therapy. Concerns with the therapy included anxiety about completing CTCH tasks, fear of talking back to voices, the need for follow up and ongoing support and concerns with adaptability of the therapy.

Conclusions

Interpretation: CTCH was generally well received and the narratives validated the overall approach. Participants did not find it an easy therapy to undertake as they were challenging a persecutor they believed had great power to harm; many were concerned, anxious and occasionally disappointed that the voices did not disappear altogether. The trusting relationship with the therapist was crucial. The need for continued support was expressed.

Strengths and limitations of this study

- The study was presented in line with the Consolidated Criteria for Reporting Qualitative Research (COREQ) ensuring the explicit and comprehensive reporting of all study elements.
- Interviews and analyses were conducted by team members not involved in the delivery of the intervention or data collection for the larger RCT. However, it is not possible to rule out social desirability bias.
- The absence of participants who did not engage in therapy at all or dropped out as their views would have been valuable in highlighting difficulties with engagement and reasons for withdrawing from CTCH.

For peer review only - http://bmjopen.bmj.com/site/about/guidelines.xhtml

Background

Acting on command hallucinations in psychosis can have serious consequences for the individual and for other people and is a major cause of clinical and public concern. Many individuals who experience auditory hallucinations include commands that instruct them to do harm to self, others or objects [(1)] [(2)]. Up to half of adult psychiatric patients continue to hear voices; of these, 48% stipulate harmful or dangerous actions rising to 69% among patients in medium secure units [(1)] While it is difficult to predict individual acts of compliance, those who have complied with voices with serious harm to themselves or others within the previous 9 months are at high risk of repeating this in the following 18 months. [(3, 4)]

Using the framework of our cognitive model of voices, we have shown that it is not only the level of activity of voices, or indeed their content that drives affect and behaviour, but the nature of the relationship with the personified voice. [(5)(6)(7)] We showed that where the voice hearer believes the voice to have malevolent intent, and crucially to have the power to deliver the threat, this can motivate compliance or appeasement behavior. [(8)] These findings have been independently replicated in a forensic population. [(9)] This theoretical framework informed the development of our Cognitive Behaviour Therapy for Command Hallucinations (CTCH) designed to weaken and challenge beliefs about voices' power, thus enabling the individual to break free of the need to comply or appease and thereby reduce harmful compliance behavior and distress. [(10)(11)] CTCH is administered over a maximum period of 9 months which includes a therapeutic window up to 25 sessions. The primary aim of the therapy is to reduce the power differential between voice and voice hearer and to test out the perceived power of the voice by examining evidence for: (a) the voice hearer's perceived lack of control over voice activity, (b) the perceived omniscience of the voice (e.g. the apparent ability of the voice to predict the future) and (c) the perceived capacity of the voice to carry out its threats for non-compliance.

We completed the first major trial of CTCH focusing on this dimension of the voice experience, the perceived power of the voice, to reduce the motivation to comply. [(12)] The trial demonstrated that the risk of further serious compliance was high in the treatment as usual group (46%), reducing to 28% in those receiving the CTCH. The rate of consent to the trial and adherence to the CTCH were in excess of 80%, providing indirect evidence that the therapy was needed and was acceptable.

The aim of this study was to explore service user experiences of a 9-month CTCH in the context of a randomized controlled trial (SRCTN62304114) including their views on acceptability and tolerability of the intervention.

Methods

Study design and settings

This was a qualitative study embedded within a larger RCT comparing CTCH and treatment as usual with treatment as usual alone. The trial involved 197 eligible participants from 3 UK centres. Participants were assigned to the intervention or control arm of the trial in a 1:1 ratio. The treatment period was a maximum of 9 months and follow up occurred at 9 and 18 months after randomization. Participants were eligible if they met all of the following criteria: had International Statistical Classification of Diseases and Related Health Problems, 10th Revision (ICD-10),

For peer review only - http://bmjopen.bmj.com/site/about/guidelines.xhtml

schizophrenia, schizoaffective (F20, 22, 23, 25, 28, 29), or mood disorders (F32),19 and were under the care of a clinical team; were aged 16 years and older; had a history of harmful command hallucinations for at least 6 months with recent (< 9 months) history of harm to self or others, or major social transgressions as a result of the commands (full or incomplete compliance); or had harmful command hallucinations whereby the individual was distressed and appeasing the powerful voice. Individuals with a primary diagnosis of addictive disorder, an organic impairment or insufficient command of English were excluded from the study. The protocol for the cognitive therapy, described in Meaden et al [10] allows the treatment to be tailored to the individual. The intervention was described in the main trial publication , with illustrative cases written up in our manual [10][11] Ethical approval for the study was provided by The West Midlands Research Ethics Committee (number 06/MRE07/71).

Research team and reflexivity

The interviews were conducted by one of the co-applicants (HL, deceased) and two research assistants (KP and LB), all unknown to participants, and examined the thoughts, concerns and experiences of the CTCH in an open manner. Therapists did not conduct the interviews nor were they present for them. The interviews continued until the participant felt they had nothing more to say in response to the framework questions proposed to them (probe questions). The interviews each lasted for approximately half an hour. Interviews were coded by two raters not involved in the trial. We believe therefore that the data collected here were representative of those receiving therapy and interpreted without bias.

Patient and public involvement

Our progenitor paper [3] interviewed patients about what was troublesome about voices and what they wanted to change; command hallucinations emerged as a frequent issue. The intervention was developed in 2006 [11] in conjunction with patients, who were consulted on outcomes and process. This paper further assesses patients' views on the therapy including the burden of the intervention. A service user in research group based in Birmingham (SURESEARCH) was consulted on the research question when the study was designed (2005) and a nominee invited to the Trial Steering Committee. Patients were not involved in recruitment or conduct of the study. Participants were not informed directly of the trial outcome but were able to access the open access primary publication.

Sample

A sample of 25 participants was drawn, who were selected randomly by number generator, from the total of 98 receiving the therapy in the trial (25.5%). These included 8 from the Birmingham site, 10 from London and 7 from Manchester, with a range of ages and genders included. The sample included participants who had completed all of the sessions as part of the CTCH program, and who consented to be interviewed and recorded for the purposes of this study. Interviews were carried out until theoretical saturation was achieved with no new themes emerging; this was achieved at 25 interviews. Participants were assured that the interviews would be anonymous and individual views of therapists would not be communicated to the therapists.

Data collection and analysis

For peer review only - http://bmjopen.bmj.com/site/about/guidelines.xhtml

Face-to-face semi-structured qualitative interviews were carried out at the sites where therapy took place, which included service bases and participants' homes. Each interview was audio recorded, transcribed verbatim (by PRSL) and checked for accuracy. Personal details were removed to preserve participant anonymity. Field notes were written in the form of memos. The study is presented in line with the Consolidated Criteria for Reporting Qualitative Research (COREQ) [13] and the Standards Reporting Qualitative Research (SRQR)[14].

The interviews were structured but flexible in allowing follow up to participant supplied information. They focused on a brief history of the patients' difficulties and symptoms, followed by discussion of the therapy they had received, and the positive and negative aspects of receiving the therapy as felt by the participants. The interview guide included the following probe questions:

Do you feel that the therapy helped you? Why do you feel that that session (which participant mentioned) was (un)helpful for you?

Was the therapy acceptable for you?

How was your relationship with [the therapist]?

Did you feel the therapist understood what it was like to hear voices?

How was the length of the sessions and the program as a whole?

What was the most helpful session? What was the least helpful session?

Do you feel the therapy made a difference at all?

The interviews were analysed using adapted grounded theory [15]. Theories were generated from the data. Evidence of dissonance was sought throughout and theories were altered to accommodate this. Quotations have been chosen to be representative of the data collected. Each transcript was read and reread by LM and EE, until saturation of data was reached, and no new themes emerged.

There was line by line thematic analysis and discussion on any discourse present in the analysis of the themes. Words, ideas and reported experiences in the participants' texts was completed with an inductive approach and no pre-existing framework for the ideas. Agreement between LM and EB was high when it was discussed at regular meetings. Thematic coding was grouped into key emergent themes, which we present as findings in this paper.

Findings

The sample consisted of 25 participants, 15 of whom were males. The mean age of the sample was 42 years, and 68% were White British. Thirty-six per cent (36%) of the sample was diagnosed as 'unspecified psychosis', 56% as 'Schizophrenia' and 8% as Schizo-affective disorder. The demographic characteristics of the sample are presented in Table 1.

Table 1 about here.

For peer review only - http://bmjopen.bmj.com/site/about/guidelines.xhtml

Two superordinate themes were identified: participants' views about the aspects of CTCH they found most helpful and participants' concerns with therapy. The themes (and sub-themes) are presented below and supported by illustrative quotations from the transcripts.

Helpful aspects of CTCH

The components of CTCH participants reported finding most helpful in tackling their mental health problems were related to:

(i) Feeling more in control

Several individuals discussed one of the outcomes of the therapy as gaining more control over the voices and being able to cope better with their mental health problems and the impact these had on their lives. Gaining control over the voices was one of the main components and aims of the intervention and in our previous work we were able to show that participants who received the CTCH intervention felt more powerful, than those in the control group, in their ability to withstand and mitigate the threat received by their voices.

'I'm able to control them [the voices] a bit more.' (F52BIR104)

"I cope better now because I listen to what [the therapist] said." (F67LON013)

"I feel like I've got control of them [the voices]." (M30BIR125)

(ii) Challenging the power and omniscience of the voices

Participants spoke about how being encouraged to reflect on evidence for voices' power and omniscience particularly helped them during the therapy. Using examples from everyday life, therapists and participants worked collaboratively to show that voices made empty threats which subsequently led participants to realise that "*the problems are all inside me. They are not out there*", as one participant noted. The therapy aimed to build on participants' own strengths to face up to the voices, and so make the voices less fearful. As indicated below, this technique was reported as particularly useful by the participants.

'She said, if we put the papers down and let's see if they come in and take them, and I said they didn't. [In response to patients belief that the voices would take her therapy notes from the therapist to find out what she had told others of the voices] I think that helped, you know. It just gives me a bit of support that they're lying. They are just talking.' (F67LON013)

'She [the therapist] was pregnant and [the voice] says that something will happen to her baby. And she had the baby and nothing happened, and that was really a good reassurance that [the voice] is just in my mind and not real. [The therapist] says he [the voice] could bring it on, that it won't hurt her and she was sure the baby would be alright. I know he can't do things physically, just mentally.' (F57BIR094)

'[The voice] is just in my mind and not real.' (F57BIR094)

For peer review only - http://bmjopen.bmj.com/site/about/guidelines.xhtml

(iii) Following a structured approach

Participants described how taking a structured approach to the therapy sessions helped them get an overview of the therapy in the context of a complex and confusing experience. Some participants specifically commented that understanding the triggers for their voices helped to gain mastery, as illustrated by the quotes below.

'It was very structured; all the time we had an agenda which was good' (F42LON077)

'She gave me some information afterwards…I could remember the session where she was helping me through this.' (F48LON085)

"Connecting things together…change your pattern and change your ways.' (F56LON009)

'She drew me a diagram between the bad thoughts and the good thoughts, and how the brain works and that diagram was helpful.' (F48LON085)

(iv) Normalisation and mainstreaming of the experience of voices

Many participants described how the acceptance of voice hearing reduced feelings of isolation and demonstrated through the experience of others that control was possible. Normalisation of the voice appeared to reduce the 'specialness' of the voice, as well as offering support to the voice hearer. Participants commented how finding out that hearing voices is common and "*can happen to most people*" (M25MAN062) was empowering and helped them feel better about themselves.

'[The therapist] was like 'oh it's common and can happen to most people.' I wasn't aware of that… She was telling me how the voices can be controlling and stuff.' (M25MAN062)

'[watched a DVD called 'am I normal' by the BBC, suggested by therapist] people that either hear voices or have heard voices and they're back in work, or they're living some kind of normal life or they're in respected positions, but they still hear voices. That was helpful; let me feel that I wasn't alone. I felt a little bit better in myself.' (M53LON001)

(v) The value of peer support

Participants highlighted that provision of peer support alongside the therapy was beneficial in normalizing their experiences, supporting them with challenging or high risk situations such as going on assignments outside, and encouraging them in engaging with difficult aspects of the therapy such as challenging the power of voices. In particular, participants felt that peer support helped them feel included and listened to; empowered them to help others, or as one participant said "give something back"; and also benefited their social life.

'The family and people where I'm living now in support housing [listen to me]. We talk about the voices. They listen to me and talk to me like, saying things aren't going to happen while you're living here.' (M30BIR126)

For peer review only - http://bmjopen.bmj.com/site/about/guidelines.xhtml

‘I’m going to a place on Tuesday, and you talk about the issues you wanna talk about. They’re there to listen.’ (M41LON101)

‘She [the therapist] put me in touch with the healing arts team, and another place called [an arts based support group for people with mental health issues] …a couple of times we went outside.’ (M53LON072)

[Patient in conversation with another patient with psychosis] ‘I just told her it gets better and he won’t win. Even though you won’t feel like it now, he won’t win. [Interviewer asks if she recommended speaking back to the voices, patient responds] ‘yeah tell them to get lost.’ (F25MAN067)

‘I really wanna do something, like give back what they give you. Take it and help other people.’ (F40MAN079)

‘I [the participant] gave her [the therapist] an example on how to cope with voices.’(M37BIR013)

Concerns with therapy

Participants also expressed several concerns with the therapy. These were mainly associated with the experience of persecutory feelings interfering with the therapy process, for example concerns about the therapist and their intentions; but also receptiveness to accepting psychological therapy. Adapting the therapy to the individual and reassurance regarding the lack of real world power of the voices were reported as helping with the tolerability of the therapy.

(i) Fear of therapy

Many participants described fear of the therapy arising from a perceived lack of ability to perform the tasks requested as part of the therapy, such as talking back to the voices. This could be related to lack of self–confidence which seemed to be more an issue at the early stages of the therapy yet caused significant distress to participants. A commonly reported concern was talking about the voices to the therapist due to fear about what the voices might do or say. For some individuals, the voices spoke directly about the therapist and instructed the individual not to reveal their conversations.

‘I was frightened about starting the therapy, because I didn’t know quite what to expect…. [During the therapy] I felt unsure whether I was answering them [the questions] correctly.’ (F48LON085)

‘They tell you not to talk about them…so you’re frightened to talk to someone about it.’ (F48LON100)

‘It’s quite frightening to try and explain these voices.’ (F67LON013)

(ii) Challenges to established patterns of thinking and behaviour

Some participants described feeling upset at times when asked to think through and challenge long-established beliefs about their voices and their behaviour in response to the voices. For example,

For peer review only - http://bmjopen.bmj.com/site/about/guidelines.xhtml

one participant described finding it very difficult to talk back to the voices following the therapist's suggestion. Trying out new ways of coping, for example testing the voice as a way of challenging participants' entrenched beliefs about the voices and their associated power was also something participants found challenging. In some cases there was resistance to some of the new suggestions and ways of coping. One participant said:

"'I got my own way of coping with things and she was trying to introduce new ways and I found it hard to try to practice them coz I was used to thinking a certain way.' (M50MAN081)"

This resistance could be related to perceived lack of self-confidence and the fear of therapy discussed previously but also highlights other concerns such as participants' fear at changing the strategies they already have for dealing with the voices. Personalising aspects of the therapy to address such concerns would be necessary, as therapy needs to take into account an individual's beliefs, fears and coping methods to plan a way forward.

'It was upsetting at times, I would leave here in floods of tears at times, but I carried on.'(F48LON085)

'[the therapist suggested the patient] should talk back to them, try and reason with them, but I didn't think that was a very good idea because then I'm being pulled into their games.' (F27LON105).

(iii) Building trust with the therapist

Many participants described how building a trusting relationship with the therapist was difficult as new and sometimes challenging ideas were discussed during therapy which some individuals felt placed them at risk from the voices. Building trust with the therapist was perceived by participants to be important in allowing them to feel confident to change established patterns of thinking and behavior. Participants explained how this did not happen overnight and a collaborative effort from the therapist and individual was necessary to achieve that. Some described how their therapist worked practically alongside them in a facilitating role, while others discussed having the therapy in an environment where they felt safe.

'It took me a little while to build up the trust [with the therapist].' (F48LON100).

'I was very frightened about leaving the hospital and on just one occasion she came and took the bus with me part way. She walked me to the bus and got on the bus with me, and I found that very supportive, and it gave me the encouragement to be able to leave the session and do it on my own. ' (F42LON077).

'She [the therapist] helped me start going places where there's other people.' (F25MAN067)

'And then we started going [to have the therapy at] my Grans and it was alright…. Because I feel safe at my Grans.' (F25MAN067)

(iv) Need for better personalisation of therapy

While some participants were pleased that the therapy and the therapists were sensitive to the individual and their circumstances, including any fears and concerns, others voiced concerns that the

For peer review only - http://bmjopen.bmj.com/site/about/guidelines.xhtml

therapy was not adaptable enough and provided some examples of how the content, duration and tasks of the therapy could be tailored to the individual's needs and preferences.

'It [the therapy] should have gone to a practical therapy. Actually confront the fears together and she could go through what I was thinking at certain points. Actually go to the public places.' (M50MAN081).

'[in response to is there anything you would change] The sessions should last longer sometimes. Or shorter sometimes. [So be more flexible?] Yeah. It was how much it applied to you.' (M46MAN076)

'She gave me things to do, like do you have any hobbies or something to take your mind off…. I was looking at a book I just couldn't read…. She said to make some noise like 'mmmmh' to like, to stop it……I felt silly doing that in the living room and my parents were [enquiring why the patient was making the noise]'(M25MAN062)

(v) Expectations of therapy: control vs cure

Participants' expectations of therapy i.e. their beliefs about the consequences of receiving treatment, such as that the treatment will lead to improvement, was a recurrent issue. Some participants reported being disappointed that the therapy did not have the impact they had expected, for example, it did not make the voices go away or as one participant said '*the magic never seemed to start*'.

'I just kept thinking, when's the magic going to start.' (M50MAN081)

'They've [the voices have] not gone away completely. You know unfortunately I thought they would but they haven't.' (F42LON077)

Discussion

In this study, we explored service user experiences of a 9-month cognitive behavioural therapy for command hallucinations in the context of a randomized controlled trial including, their views on acceptability and tolerability of the intervention.

The study was presented in line with COREQ ensuring the comprehensive reporting of all study elements. Interviews and analyses were conducted by team members not involved in the delivery of the intervention or data collection for the larger RCT. Indeed, therapists were not involved in the qualitative study nor were present during the interviews. A limitation of this study was the absence of participants who did not engage in therapy at all or dropped out as their views would be valuable in highlighting difficulties with engagement and reasons for withdrawing from CTCH.

Few CBT studies in psychosis have sought systematically to document participants' experience of therapy [(16,17)]. A recent synthesis of existing qualitative studies concluded that an alliance between service user and therapist, clear steps in facilitating change, and the challenges of applying CBTp (cognitive behavioral therapy for psychosis) are the main reported experiences of users [(17)]. In this study too, participants highlighted the importance of the therapist-service user relationship in distancing from voices' power, the value of structure in the therapy, and how the CTCH could be

For peer review only - http://bmjopen.bmj.com/site/about/guidelines.xhtml

difficult and distressing for participants. In addition, the present study also documented how normalising the voice experience served to gain intellectual mastery of it (i.e. voices were no longer 'special' or 'mysterious') and many highlighted the need for longer-term, preferably and peer support.

The components of the CTCH service users found most useful was gaining more control over the voices and challenging the power of voices to act on their supposed malevolent intent. The words 'control' and 'cope' cropped up frequently in the narratives of participants. Many valued the structured approach to examining long-held beliefs and practices; for example clarity about the triggers for voices helped to achieve intellectual mastery of the experience and as a basis to challenge the power of the persecutor behind the voice. Others described the value in normalising the experience; this characterisation as an everyday event served as an antidote to the 'special' place of the voice and indeed of the voice hearer within it. Some argued that this could have gone further with improved peer support and validation. Trusting the therapist was brought up in many of the narratives. This is understandable from the client's point of view as from their perspective, the voice is embodied with major threats, in some cases life-threatening, and hence trusting the therapist's motives and ability was seen as crucial. Participants recounted many examples of the lengths therapists went to achieve this; accompanying them on assignments in supposed high risk situations was often described and valued by the participants.

Some concerns with the therapy, albeit minor, were also expressed and these centered on the experience of persecutory feelings interfering with the therapy process, for example concerns about the therapist and their intentions; but also, receptiveness to accepting psychological therapy. Many participants reported feeling fearful of the therapy and found the process of facing up to the power of voices at times distressing. Simply articulating these complex experiences was sometimes profoundly difficult for individuals. In some cases, the voices attempted to exert counter-control by questioning the therapist and commanding the individual to disengage. This is perhaps where the suggestion for more peer support from participants would have been beneficial. It was also noted to be beneficial for the therapist to provide reassurance in the form of reiterating that the voices cannot cause affect in the real world. Some participants expressed concerns that the therapy *"did not get rid of the voices".* Managing expectations of the therapy is crucial for therapeutic success and may influence the relationship between therapist and client [(18)]). Therapists have a key role in communicating clearly at the outset -and throughout the therapy- the intended changes and benefits of the therapy. This will allow a shared understanding of how therapy will proceed, facilitating engagement and rapport. Nonetheless, none of the individuals we spoke to expressed strong adverse experiences of the therapy and as we highlight, few dropped out (n=7).

Implications for practice

The views of participants captured in this study combined with the high level of completion of the therapy and retention in the trial, indicates that the CTCH is an acceptable intervention and generally well tolerated. Clinicians working with individuals experiencing harmful voices should be mindful of asking, as part of their assessment, about the perceived power of the voice relative to the individual. This is important as our work [(19)] has shown that the perceived power of voices to threaten the individual, was the best predictor of harmful compliance to voices.

For peer review only - http://bmjopen.bmj.com/site/about/guidelines.xhtml

Nonetheless, clinicians should also be mindful of challenges reported by individuals in relation to the CTCH e.g. not being an easy therapy to undertake; fear that the approach could increase the perceived threat from voices, and maximize efforts to build alliance. We recommend peer-support as a useful addition to this therapy, particularly in providing support following its conclusion but also in providing help during difficult periods when the voice's power is confronted and to further normalize the experience (an approach recently highlighted as a '*promising practice'* in the 2014 NICE guidance for Schizophrenia). [20] We also recommend to therapists implementing this approach to make it clear that the intervention is not designed to eliminate voices rather build on individuals' own strengths to face up to the power of the voice, to make them less fearful and place them in the 'driving seat'.

For peer review only

For peer review only - http://bmjopen.bmj.com/site/about/guidelines.xhtml

Funding: This work was supported by the UK Medical Research Council (G0500965) and the National Institute for Health Research.

Competing interests: None declared.

Patient consent: Obtained.

Contributors: MB, EP, SL, TW, NT, LD, and GD designed the randomized controlled trial. MM was the trial manager. MB developed the cognitive therapy for command hallucinations. MB and AM designed and led the training for cognitive therapy for command hallucinations, and AM and EP led the supervision process and the rating of therapy tapes. LM and MM analysed the data and with MB wrote the first draft of the manuscript. LM and MM redrafted the manuscript. All authors read and approved the final manuscript. MB had full access to all the data in the study and had final responsibility for the decision to submit for publication.

Data sharing statement: Anonymised interview transcripts are available for further analysis under the University of Warwick data sharing policy [http://www2.warwick.ac.uk/services/ris/research_integrity/code_of_practice_and_policies /research_code_of_practice/datacollection_retention/research_data_mgt_policy]. Please contact MB.

Acknowledgements: The late Professor Helen Lester conducted the interviews and had oversight of their transcription.

Acknowledgements: MB is part-funded by the National Institute for Health Research (NIHR) Collaborations for Leadership in Applied Health Research and Care West Midlands. This paper presents independent research and the views expressed are those of the author(s) and not necessarily those of the NHS, the NIHR or the Department of Health and Social Care. TW would like to acknowledge the support her NIHR Senior Investigator award and TW and EP the NIHR Biomedical Research Centre at the South London and Maudsley NHS Foundation Trust and King's College London.

For peer review only - http://bmjopen.bmj.com/site/about/guidelines.xhtml

References

1. Shawyer F, Mackinnon A, Farhall J, Trauer T, Copolov D. (2003). Command hallucinations and violence: implications for detention and treatment. *Psychiatry, Psychology and Law*, 10:97-107.
2. Appelbaum PS, Robbins P., Monahan J. (2000). Violence and delusions: data from the MacArthur Violence Risk Assesment. *American Journal of Psychiatry,* 157, 566-572
3. Chadwick, P., Birchwood, M. (1994). The omnipotence of voices: a cognitive approach to auditory hallucinations. *British Journal of Psychiatry*, 164, 190-201.
4. Trower P, Birchwood M, Meaden A, Byrne S, Nelson A, Ross K. (2004). Cognitive therapy for command hallucinations: randomised controlled trial. *British Journal of Psychiatry,* 184 (4) 312-320;
5. Birchwood, M., Gilbert, P., Gilbert, J., Trower, P., Meaden, A., Hay, J., Murray, E. Miles, J.N.V. (2004). Do interpersonal schema influence the relationship with the dominant 'voice' in schizophrenia? A comparison of three models. *Psychological Medicine,* 34, 1571-1580.
6. Connor, C., Birchwood, M. (2013). Power and perceived expressed emotion of voices: their impact on depression and suicidal thinking in those that hear voices. *Clinical Psychology and Psychotherapy, 20(3):199-205.*
7. Byrne S, Trower P, Birchwood M. (2003). Command Hallucinations: cognitive theory, cognitive therapy and research. *Journal of Cognitive Psychotherapy*, 67-84.
8. Bucci,S., Birchwood,M., Twist,L., Tarrier,N., Emsley,R. and Haddock, G (2013) Predicting compliance with command hallucinations: Anger, impulsivity and appraisals of voices' power and intent, Schizophrenia Research , Volume 147 , Issue 1 , 163 - 168
9. Fox J, Gray N, Lewis N. (2004). Factors determining compliance with violent content: the role of social rank, perceived power of the voice and voice malevolence. *Journal of Forensic Psychiatry and Psychology,* 15:511-31.
10. Meaden A, Keen N, Aston R, Barton K, Bucci S. *Cognitive Therapy for Command Hallucinations: An advanced practical companion*. Routledge Taylor and Francis Group, London and New York, 2013,

For peer review only - http://bmjopen.bmj.com/site/about/guidelines.xhtml

11. Byrne S, Birchwood M, Trower P, Meaden A. *A Casebook of cognitive behaviour therapy for command hallucinations. A social rank theory approach*. London & New York: Routledge; 2006.
12. Birchwood M, Michail M, Meaden A, Tarrier N, Lewis S, Wykes T et al (2014). Cognitive behavioural therapy to prevent harmful compliance with command hallucinations (COMMAND): a randomised controlled trial. *Lancet Psychiatry*, 1: 23-33.
13. Tong A, Sainsbury P, Craig J. (2007). Consolidated criteria for reporting qualitative research (COREQ): a 32-item checklist for interviews and focus groups. International Journal for Quality in Health Care, 19:349–57
14. O'Brien BC, Harris IB, Beckman TJ, Reed DA, Cook DA (2014). Standards for reporting qualitative research: a synthesis of recommendations. *Academic Medicine*, Vol. 89, No. 9 / Sept 2014 DOI: 10.1097/ACM.0000000000000388
15. Birks M, Mills J. *Grounded Theory: A Practical Guide*, Sage 2011, London.
16. Kilbride M., Byrne R., Price J., Wood L., Barratt S., Welford M., Morrison A.P. (2012). Exploring service users' perceptions of cognitive behavioural therapy for psychosis: a user led study. *Behavioural and Cognitive Psychotherapy*, 41 (1), 89-102
17. Wood L., Burke E., Morrison A, (2015). Individual Cognitive Behavioural Therapy for Psychosis (CBTp): A Systematic Review of Qualitative Literature *Behavioural and Cognitive Psychotherapy*, 43, 285–297
18. Greenberg RP, Constantino MJ, Bruce N. (2006). Are patient expectations still relevant for psychotherapy process and outcome? *Clinical Psychology Review*, 26: 657–678
19. Birchwood M., Dunn G., Meaden A., Tarier N., Lewis S., Wykes T., Davies L., Michail M., Peters E. (2017). The COMMAND trial of cognitive therapy to prevent harmful compliance with command hallucinations: predictors of outcome and mediators of change. Psychological Medicine. https://doi.org/10.1017/S0033291717003488
20. National Institute for Clinical Excellence. *Psychosis and schizophrenia in adults: prevention and management,* NICE guidelines [CG178], February 2014

For peer review only - http://bmjopen.bmj.com/site/about/guidelines.xhtml

Table 1. Participant characteristics.

Identifier	Gender	Age	Ethnicity	Diagnosis
F56LON009	Female	56	British	Unspecified psychosis
F67LON013	Female	67	British	Unspecified psychosis
M53LON072	Male	53	Other-white background	Schizophrenia
F42LON077	Female	42	Caribbean	Schizo-affective disorder
F48LON085	Female	48	British	Unspecified psychosis
M18LON099	Male	18	British	Unspecified psychosis
F48LON100	Female	48	British	Unspecified psychosis
M41LON101	Male	41	British	Schizophrenia
F27LON105	Female	27	British	Schizophrenia
M53LON001	Male	53	Otherblack background	Schizophrenia
M39BIR093	Male	39	British	Schizophrenia
F57BIR094	Female	57	British	Schizophrenia
M55BIR016	Male	55	Irish	Schizophrenia
M30BIR125	Male	30	Caribbean	Unspecified psychosis
M35BIR126	Male	35	British	Unspecified psychosis
M37BIR013	Male	37	Caribbean	Schizophrenia
F52BIR104	Female	52	British	Schizo-affective disorder
M34BIR045	Male	34	British	Schizophrenia
F25MAN067	Female	25	British	Unspecified psychosis

For peer review only - http://bmjopen.bmj.com/site/about/guidelines.xhtml

M25MAN062	Male	25	British	Schizophrenia
M31MAN066	Male	31	British	Unspecified psychosis
M46MAN076	Male	46	British	Schizophrenia
M47MAN073	Male	47	Other-black background	Schizophrenia
F40MAN079	Female	40	African	Schizophrenia
M50MAN081	Male	50	British	Schizophrenia

For peer review only

For peer review only - http://bmjopen.bmj.com/site/about/guidelines.xhtml

Standards for Reporting Qualitative Research (SRQR)*

http://www.equator-network.org/reporting-guidelines/srqr/

Page/line no(s).

Title and abstract

**Title** - Concise description of the nature and topic of the study Identifying the study as qualitative or indicating the approach (e.g., ethnography, grounded theory) or data collection methods (e.g., interview, focus group) is recommended	1
**Abstract** - Summary of key elements of the study using the abstract format of the intended publication; typically includes background, purpose, methods, results, and conclusions	2

Introduction

**Problem formulation** - Description and significance of the problem/phenomenon studied; review of relevant theory and empirical work; problem statement	3
**Purpose or research questio**n - Purpose of the study and specific objectives or questions	3

Methods

**Qualitative approach and research paradigm** - Qualitative approach (e.g., ethnography, grounded theory, case study, phenomenology, narrative research) and guiding theory if appropriate; identifying the research paradigm (e.g., postpositivist, constructivist/ interpretivist) is also recommended; rationale**	3
**Researcher characteristics and reflexivity** - Researchers' characteristics that may influence the research, including personal attributes, qualifications/experience, relationship with participants, assumptions, and/or presuppositions; potential or actual interaction between researchers' characteristics and the research questions, approach, methods, results, and/or transferability	4
**Context** - Setting/site and salient contextual factors; rationale**	3-4
**Sampling strategy** - How and why research participants, documents, or events were selected; criteria for deciding when no further sampling was necessary (e.g., sampling saturation); rationale**	4
**Ethical issues pertaining to human subjects** - Documentation of approval by an appropriate ethics review board and participant consent, or explanation for lack thereof; other confidentiality and data security issues	4
**Data collection methods** - Types of data collected; details of data collection procedures including (as appropriate) start and stop dates of data collection and analysis, iterative process, triangulation of sources/methods, and modification of procedures in response to evolving study findings; rationale**	4-5

For peer review only

For peer review only - http://bmjopen.bmj.com/site/about/guidelines.xhtml

**Data collection instruments and technologies** - Description of instruments (e.g., interview guides, questionnaires) and devices (e.g., audio recorders) used for data collection; if/how the instrument(s) changed over the course of the study	4-5
**Units of study** - Number and relevant characteristics of participants, documents, or events included in the study; level of participation (could be reported in results)	5
**Data processing** - Methods for processing data prior to and during analysis, including transcription, data entry, data management and security, verification of data integrity, data coding, and anonymization/de-identification of excerpts	4-5
**Data analysis** - Process by which inferences, themes, etc., were identified and developed, including the researchers involved in data analysis; usually references a specific paradigm or approach; rationale**	4-5
**Techniques to enhance trustworthiness** - Techniques to enhance trustworthiness and credibility of data analysis (e.g., member checking, audit trail, triangulation); rationale**	5

Results/findings

**Synthesis and interpretation** - Main findings (e.g., interpretations, inferences, and themes); might include development of a theory or model, or integration with prior research or theory	5
**Links to empirical data** - Evidence (e.g., quotes, field notes, text excerpts, photographs) to substantiate analytic findings	5-10

Discussion

**Integration with prior work, implications, transferability, and contribution(s) to the field -** Short summary of main findings; explanation of how findings and conclusions connect to, support, elaborate on, or challenge conclusions of earlier scholarship; discussion of scope of application/generalizability; identification of unique contribution(s) to scholarship in a discipline or field	10-11
**Limitations** - Trustworthiness and limitations of findings	2

Other

**Conflicts of interest** - Potential sources of influence or perceived influence on study conduct and conclusions; how these were managed	12
**Funding** - Sources of funding and other support; role of funders in data collection, interpretation, and reporting	12

*The authors created the SRQR by searching the literature to identify guidelines, reporting standards, and critical appraisal criteria for qualitative research; reviewing the reference lists of retrieved sources; and contacting experts to gain feedback. The SRQR aims to improve the transparency of all aspects of qualitative research by providing clear standards for reporting qualitative research.

For peer review only - http://bmjopen.bmj.com/site/about/guidelines.xhtml

**The rationale should briefly discuss the justification for choosing that theory, approach, method, or technique rather than other options available, the assumptions and limitations implicit in those choices, and how those choices influence study conclusions and transferability. As appropriate, the rationale for several items might be discussed together.

Reference:

O'Brien BC, Harris IB, Beckman TJ, Reed DA, Cook DA. **Standards for reporting qualitative research: a synthesis of recommendations.** *Academic Medicine*, Vol. 89, No. 9 / Sept 2014 DOI: 10.1097/ACM.0000000000000388

For peer review only
